# Store-operated calcium signaling modulators improve a dendritic cell-based vaccine against melanoma

**DOI:** 10.1080/2162402X.2026.2723541

**Published:** 2026-08-28

**Authors:** María Cruz Cobo, Laure Garnier, Marion Mandavit, Maral Azam, Isabelle Dentand Quadri, Julien Angelillo, Dale Brighouse, Mathilde Bausart, Valérie Dutoit, Achille Schild, Valeria M. Oliva, Martin Lochner, Bénédicte Manoury, Stéphanie Hugues, Paula Nunes-Hasler

**Affiliations:** a Geneva Center for Inflammation Research, Faculty of Medicine, Pathology and Immunology Department, University of Geneva, Geneva, Switzerland; b Translational Research Centre in Onco-Hematology, Faculty of Medicine, Pathology and Immunology Department, University of Geneva, Geneva, Switzerland; c Brain Tumor and Immune Cell Engineering Laboratory, Translational Research Center in Oncohaematology, Faculty of Medicine, Department of Medicine, University of Geneva, Geneva, Switzerland ; and Swiss Cancer Center Léman (SCCL), Switzerland; d Institute of Biochemistry and Molecular Medicine, University of Bern, Bern, Switzerland; e Institut Necker Enfants Malades, INSERM U1151-CNRS UMR 8253, Faculté de Médecine Necker, Université Paris Cité, Paris, France

**Keywords:** Antigen presentation, DC vaccine, store-operated calcium entry, melanoma, immunotherapy, cell-based therapy

## Abstract

Dendritic cells (DCs) are crucial for eliciting cytotoxic CD8^+^ T cell responses against intracellular pathogens, viruses, and cancer. DC-based vaccines can stimulate both cytotoxic and humoral immunity, as well as memory against cancer. However, efficacy has remained low, leaving room for improvement. Our previous work using conditional *Stim1* knock-out mice highlighted the importance of store-operated calcium entry (SOCE) in promoting DC migration, phagosome maturation, and DC-driven CD8^+^ T cell responses. Yet its potential as a target to improve DC-mediated immune stimulation remains incompletely understood. We hypothesized that transient pharmacological modulation of SOCE signaling could boost DC-mediated T cell activity. Here, a targeted screen of SOCE modulators identified two compounds, thapsigargin and *para*-bromo-2-aminoethyl diphenylborinate, that improved T cell proliferation, IL-2, and IFNG secretion in mouse and human DC:T cell co-cultures. In a B16 melanoma model, DC vaccines boosted with either compound reduced tumor growth by 50% and doubled the median survival benefit, increasing infiltration of activated CD8^+^ T cells, CD4^+^ T, natural killer, CD80^+^ B cells, and M1 macrophages. Neither compound affected the surface expression of MHC-I-peptide complexes, MHC-II, CD86, nor DC migration, although thapsigargin promoted antigen retention. Bulk RNA sequencing revealed instead broad changes in pathways regulating secretion, where cytokine array analysis confirmed both compounds boosted secretion of several immunomodulatory factors. Together, this work identifies SOCE as a druggable axis that may be exploited to boost DC-mediated T cell activation and improve DC-based anticancer therapies.

**Summary:**

Cruz Cobo et al. identify small molecules that reprogram dendritic cells toward an immuno-stimulating secretory phenotype, revealing endoplasmic reticulum calcium signaling as a druggable axis to boost vaccine-based and T cell-mediated cancer immunotherapies. The study links broad changes in calcium-induced secretion to improved immune activation and therapeutic potency.

## Introduction

Dendritic cells (DCs) are phagocytic innate immune cells specialized in antigen acquisition, processing, and presentation. The high expression of both major histocompatibility I and II complexes (MHC-I and MHC-II) and their interactions with both T and B cells play a critical role in initiating both cytotoxic and humoral adaptive immune responses.^1^ ^,^ ^2^ In addition, through the secretion of soluble immune mediators as well as through contact-mediated interactions, they influence a large variety of immune cells, including monocytes, macrophages, and natural killer (NK) cells.^2^

DCs are heterogeneous myeloid cells of hematopoietic origin that are subdivided into several types. The major subtypes include two circulating conventional subtypes (cDC1 and cDC2) and plasmacytoid DC (pDC), derived from common DC progenitors, as well as monocyte-derived DCs (MoDCs) generated mainly during inflammation.^3^ In mice, cDC1s are generally considered the dominant DC subset mediating CD8^+^ T cell priming through antigen cross-presentation, although cDC2s have also been reported to cross-present under certain conditions.^4^ ^,^ ^5^ In humans, although cDC1s can display higher levels of cross-presentation depending on the antigen format, culture conditions, and source tissue, cDC2s and MoDCs retain the ability to cross-present.^4^ As a class, DCs have a superior ability to activate CD8^+^ T cells than other antigen-presenting cells, such as macrophages and B cells; however, the underlying molecular mechanisms are not entirely understood.^2^

Given the crucial role of CD8^+^ T cells in anti-tumor immunity and the ability of DCs to activate CD8^+^ T as well as coordinate other immune cells, DC vaccines have motivated extensive basic and clinical research for over 30 y.^6^ ^,^ ^7^ In addition, the ability of DCs to co-ordinate other tumor-reactive immune cells, including NK, CD4^+^ T, and B cells, contributes to robust and persistent anti-cancer immune responses.^1^ ^,^ ^8^ ^,^ ^9^ However, to date, while consistently showing benefits in clinical response rates^6^ ^,^ ^7^ and in some cases curative (>12 y survival) responses,^10^ DC vaccination has seldom shown large benefits in overall survival, leaving much room for improvement.^7^ Since cDC1s are rare and difficult to isolate or produce in large numbers, the majority of DC vaccines employ MoDCs that are easily obtained. However, in addition to their inefficient homing to lymph nodes, their inferior ability to activate CD8^+^ T cells is believed to be a major factor hindering the success of DC vaccines.^6^ ^,^ ^7^ As such, methods that can boost the ability of MoDCs to activate CD8^+^ T cells have the potential to improve the therapeutic efficacy of DC vaccines.

Store-operated calcium entry (SOCE) is a ubiquitous calcium signaling pathway triggered by the release of calcium stored in the endoplasmic reticulum (ER), such as during inositol trisphosphate (IP3)-mediated signaling.^11^ Loss of ER calcium is sensed by stromal interaction protein (STIM)-family transmembrane proteins. These ER-calcium binding proteins then remodel the ER and translocate to sites of tight contact between the ER and plasma membrane, where direct interaction leads to the opening of plasma-membrane resident channels of the Orai and TRPC families.^11^ SOCE can influence a large variety of functions, including gene expression, proliferation, migration, endomembrane fusion, and secretion, and is the primary calcium signaling pathway in DCs.^11^ ^,^ ^12^ Although SOCE induces cytokine secretion and affects NF-kB signaling in several immune cells,^12-14^ *Stim1*/*Stim2* double knock-out in DCs did not impair cytokine secretion or co-stimulatory molecule surface expression in response to several common stimuli.^13^ ^,^ ^15^ In contrast, our previous studies have demonstrated a critical role for STIM1 in promoting efficient DC-mediated CD8^+^ T cell activation.^15^ ^,^ ^16^ This effect was observed using siRNA-mediated STIM1 knockdown in bone-marrow-derived DCs and a cDC1 cell line, but was most pronounced in *Stim1* knockout bone marrow-derived DCs (BMDCs, generally considered similar to MoDCs).^15^ ^,^ ^16^ Here, STIM1 deletion was associated with partial defects in DC migration towards calcium-dependent chemoattractants. It also led to both potentially detrimental and beneficial changes in antigen processing pathways. Specifically, recruitment of ER membranes and the antigen-processing enzyme insulin-regulated aminopeptidase (IRAP), both important for cross-presentation,^17^ ^,^ ^18^ were reduced, while lysosome fusion, typically considered inhibitory for cross-presentation, was also decreased.^15^ ^,^ ^19^ In the present study, we sought to gain further insight into the mechanisms by which SOCE regulates DC-mediated T cell activation, as well as to investigate the translational potential of targeting SOCE to boost the efficacy of DC vaccines. A targeted screen of SOCE-modulating compounds is described, two of which were further characterized. *In vitro* and *in vivo* experiments using cell lines and *in vitro*-differentiated DCs revealed that treating DCs with SOCE-modulating compounds during antigen loading boosted CD8^+^ T cell responses. Finally, in an *in vivo* DC vaccine model, DCs treated with these compounds showed increased tumor infiltration of CD8^+^ and CD4^+^ T cells, B and NK cells, and M1 macrophages. Together, these data strongly support the translational potential of targeting SOCE to improve DC-based immunotherapy design.

## Results

### SOCE modulators boost murine DC-mediated T cell activation

**Figure 1.**
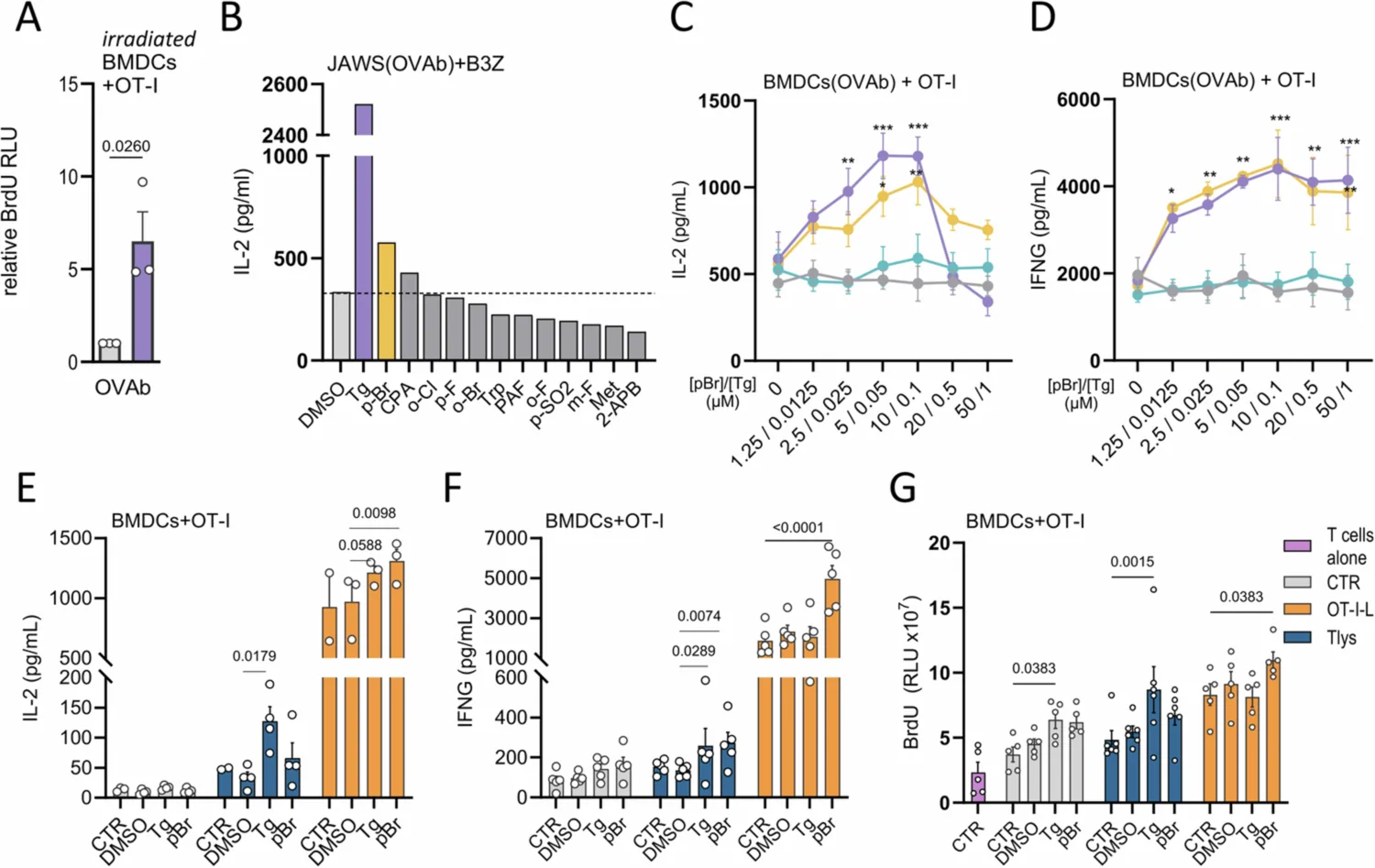
SOCE-modulating compounds boost DC-mediated T cell activation in murine DCs. (A) Proliferation of naïve OT-I CD8^+^ T cells co-cultured with irradiated LPS-matured bone marrow-derived dendritic cells (BMDCs) that were pulsed with ovalbumin-coated beads (OVAb) and either left untreated (CTR, gray) or treated with the SOCE activator thapsigargin (0.1 µM Tg, purple). Proliferation was measured by BrdU incorporation (RLU, relative luminescence units; values normalized to control, *n* = 3 independent biological samples, triplicate wells). (B) Screening of SOCE modulators in 24 h co-cultures of OVA-specific B3Z CD8? T cell hybridoma with LPS-matured, OVAb-pulsed JAWSII DCs. IL-2 secretion measured by ELISA identifies Tg (purple) and pBr (yellow) as lead compounds (single screening experiment, triplicate wells). CPA, cyclopiazonic acid; PAF, platelet-activating factor; 2-aminoethoxydiphenyl borate (2-APB) derivatives are named according to the position (*para- ortho- meta-*) of the added functional group with respect to the 2-APB parental molecule: *p*-Br, *para-*bromo; o-Cl, *ortho-*chloro; *p*-F, *para-*fluoro; o-Br, *ortho-*bromo; Trp, l-5-hydroxytryptophan; o-F, *ortho*-fluoro; *p*-SO2, (*p*-SO_2_NMe_2_) *para*-dimethylsulfamoyl; m-F, *meta*-fluoro; Met, l-methionine. See reference (22) for exact chemical structures. (C, D) Concentration-response curves of cytokine secretion following 24 h co-culture of OT-I CD8? T cells with LPS-matured BMDCs loaded with OVAb. IL-2 (C) and IFNG (D) levels were measured by ELISA following stimulation with vehicle control (DMSO, cyan), untreated control (CTR, gray), or Tg (purple) or pBr (yellow) at the indicated concentrations (µM; CTR: *n* = 2, all other conditions *n* = 3 independent biological samples, duplicate wells). (E–G) OT-I CD8? T cells were co-cultured with LPS-matured BMDCs pulsed with either B16-OVA melanoma tumor cell lysate (Tlys, blue), a long OVA-derived peptide (OT-I-L, orange), or no antigen (CTR, gray), and stimulated with Tg (0.1 µM) or pBr (10 µM). Supernatant IL-2 measured by ELISA (E; no drug CTR/DMSO/Tg/pBr: CTR/Tlys/OT-I-L *n* = 3/2/2; 4/4/3; 4/4/3; 4/4/3 independent experiments, duplicate wells) or supernatant IFNG measured by ELISA (F; *n* = 5 independent experiments for all conditions). Proliferation was measured by BrdU incorporation (G; CTR/Tlys/OT-I-L/T cells alone *n* = 5/6/5/5 independent experiments, duplicate wells). Data in A, C–G were obtained across multiple independent experimental days; antigen conditions (CTR, Tlys, OT-I-L) were included in parallel within each experiment when possible. Unequal *n* values reflect the occasional absence of specific conditions due to limited cell numbers or sample loss. Bars are means + SEM. Data in E–G were normalized by each experimental day to the mean response across all conditions measured in that experiment, and each biological replicate represents the average of technical replicate wells. Significant *p*-values were calculated using a one-sample t-test in A, or two-way ANOVA with Dunnett’s post-hoc test in C–G. In C–D, significant *p*-values are shown as *p < 0.05, **p < 0.01, ***p < 0.001. In A and E–G, exact *p*-values are indicated when significant.

In DCs, SOCE is triggered by various stimuli, including migration and phagocytosis. Ingestion of solid particles can potentiate cross-presentation, although the mechanisms are not completely understood, and may include increased antigen cytosol transfer and decreased lysosomal antigen destruction.^2^ ^,^ ^20^ ^,^ ^21^ Our previous studies showed that reducing SOCE through STIM1 depletion hindered the DC-mediated CD8^+^ T cell responses to particulate (solid) antigens, but not soluble minimal peptides, in both naïve and mature DCs.^15^ ^,^ ^16^ Thus, to determine whether SOCE activation could potentiate DC-mediated T cell activation, the SOCE-activator sarco-endoplasmic reticulum calcium ATPase (SERCA) inhibitor thapsigargin (Tg) was tested for its capacity to boost DC-triggered T cell responses. To this end, lipopolysaccharide (LPS)-matured BMDCs were exposed to ovalbumin (OVA)-coated beads (OVAb). The cells were then washed to remove Tg and irradiated to prevent BMDCs' proliferation prior to co-culture with OVA-specific OT-I CD8^+^ T cells. Indeed, BMDCs treated with Tg displayed a 6-fold increase in CD8^+^ T cell proliferation (Figure 1A). To assess whether this effect was a generalized phenomenon, we used unirradiated or immature BMDCs or the JAWSII BMDC-like cell line. DCs were pulsed with OVAb, soluble long OT-I-L peptides, or non-clarified tumor lysates (Tlys, containing solid cellular debris) of OVA-expressing B16 melanoma tumor cells. They were then co-cultured with CD8^+^ OT-I, CD4^+^ OT-II T cells, or the B3Z OVA-responsive CD8^+^ effector T cell line. The T cell response was measured as IL-2 in the supernatant at 24 h (Figure S1A–C). The enhancing effect of Tg was recapitulated in all combinations, particularly in the JAWSII/B3Z co-cultures, demonstrating that these cell lines are sensitive surrogates useful for screening SOCE modulators. Thus, 13 SOCE modulators, including Tg and recently characterized derivatives of the known SOCE modulator, 2-aminoethyl diphenylborinate (2-APB)^22^ were screened for their ability to boost DC-mediated T cell responses. The concentrations were chosen based on the published ranges for activating SOCE (Figure 1B). Tg and *para*-bromo-2-APB (pBr) (Figure S1D) induced the highest IL-2 response, and were chosen for further characterization using murine BMDCs in co-culture with naïve OT-I CD8^+^ T cells. To further characterize the activity of the compounds, the condition that induced the strongest IL-2 response (LPS-matured cells loaded with OVAb) was selected for a concentration-response analysis. IL-2 and IFNG production were both measured as indicators of T cell proliferation and activation towards a cytotoxic phenotype. The IL-2 response exhibited a hormetic concentration-response curve for both compounds (Figure 1C), whereas inhibition at higher concentrations was less evident for the IFNG response (Figure 1C, D). A more extensive analysis was conducted using these optimized concentrations by examining their effect in the context of antigen formats more typically employed in DC vaccines: OT-I-L and Tlys (Figure 1E–G). As expected, the magnitude of T cell responses was lower with these more physiological antigen formats compared to bead-associated antigens that induce phagocytosis, yet overall, both compounds improved the T cell responses to OT-I-L and Tlys stimulation, although some differences were observed. Notably, Tg induced a small increase in IFNG production in the context of Tlys, but not OT-I-L stimulation, whereas pBr did not enhance IL-2 secretion in the context of Tlys (Figure 1E, F). Furthermore, pBr slightly enhanced T cell proliferation in the context of OT-I-L but not Tlys, whereas Tg significantly boosted T cell proliferation only in the Tlys condition (Figure 1G). Notably, in these unirradiated cells, Tg also induced a small non-specific proliferation in the absence of antigen (CTR), potentially masking the effects in the context of OT-I-L (Figure 1G). These results demonstrate that the SOCE modulators Tg and pBr can enhance DC-mediated T cell activation under various conditions, with distinct effects on T cell proliferation and activation markers.

### SOCE modulators enhance *in vivo* T cell responses without impairing viability or migration

**Figure 2.**
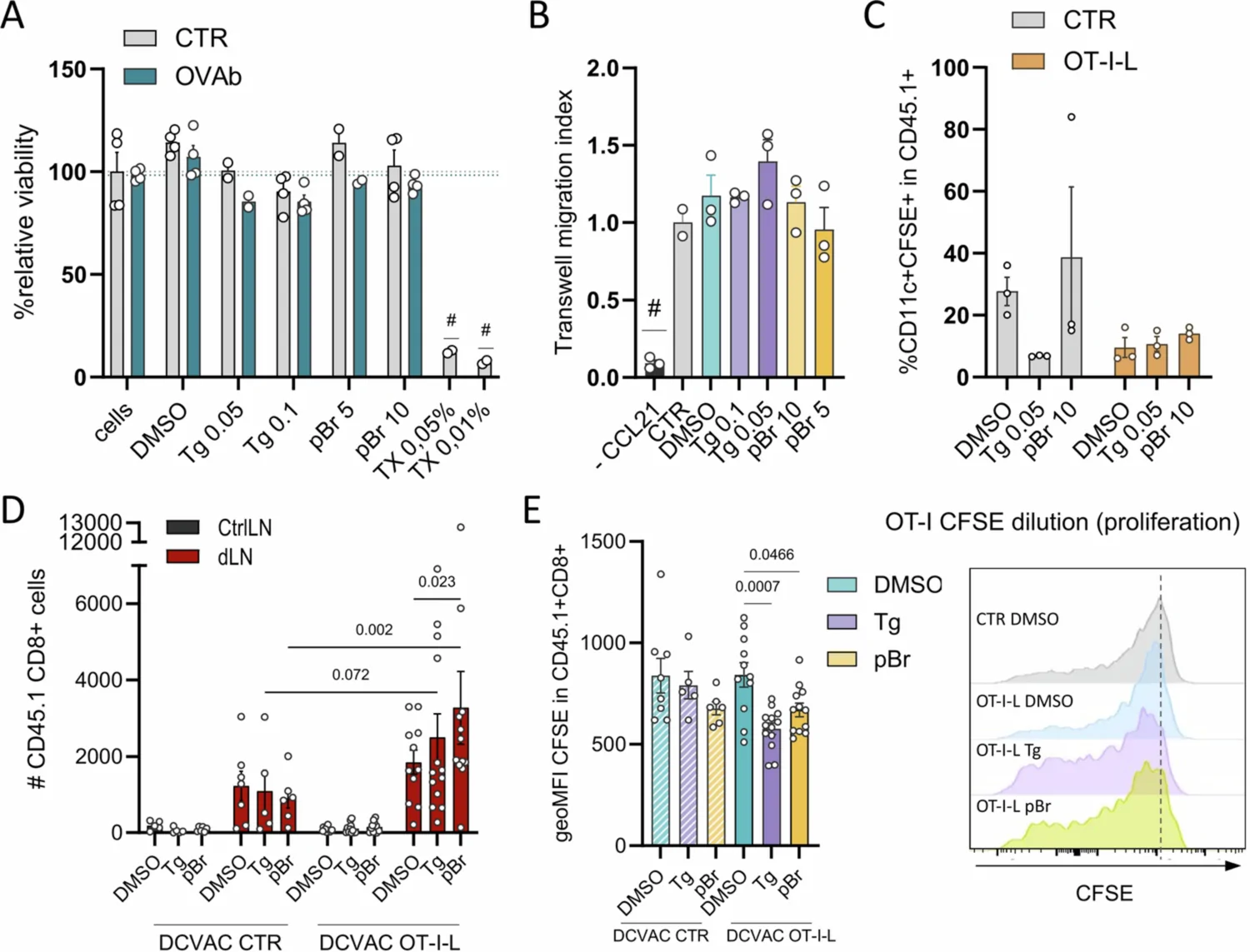
SOCE modulators boost *in vivo* T cell responses without impairing BMDC viability or migration. (A) Resazurin-based 24 h viability assay of LPS-matured, OVAb-loaded (blue) or unloaded (CTR, gray) BMDCs after 4 h exposure to SOCE modulators Tg and pBr at the indicated concentrations (µM). Triton X-100 detergent (TX) was added as a positive control. CTR/DMSO/Tg0.05/Tg0.1/pBr5/pBr10/TX0.05/TX0.01: *n* = 4/4/2/4/2/4/2/2 independent experiments, duplicate wells. Values were normalized within each experimental day and expressed as percentage relative viability compared with untreated control cells. (B) *In vitro* transwell 24 h migration towards CCL21 of LPS-matured BMDCs treated with SOCE modulators Tg (purple) or pBr (yellow) at the indicated concentrations (µM), untreated (CTR, gray), or DMSO-treated (cyan) cells, assessed by flow cytometry (FC) (-CCL21/CTR/DMSO/Tg0.1/Tg0.05/pBr10/pBr5: *n* = 3/2/3/3/3/3/3 independent experiments). Migrated cell numbers were normalized within each experimental day to the mean number of migrated cells across all conditions measured on the same experimental day, and are expressed as relative migration with respect to untreated (CTR) cells. (C) Competitive *in vivo* BMDC migration/recovery assay. Unloaded (CTR, gray) or OT-I-L-loaded (orange) LPS-matured CD45.1^+^ BMDCs labeled with CFSE and treated with SOCE modulators Tg and pBr, at the indicated concentrations (µM), and mixed 1:1 with matched untreated, unlabeled CD45.1? BMDCs before footpad injection into CD45.2 recipient mice. Migration/recovery was measured 24 h after injection as the percentage of recovered CFSE?CD45.1?CD11c? treated BMDCs in draining lymph nodes (dLN). Gating strategy is shown in Figure S2A (*n* = 3 mice). (D, E) Adoptive transfer assay: CD45.2 mice received intravenous CFSE-labeled OT-I CD8? T cells followed by footpad vaccination (DCVAC) with LPS-matured BMDCs loaded or not (CTR) with OT-I-L and treated with Tg (purple, 0.05 µM), pBr (yellow, 10 µM), or DMSO (cyan). (D) Total OT-I CD8? T cell numbers in the popliteal lymph node of the non-injected contralateral footpad (CtrlLN, dark gray) and draining popliteal lymph node (dLN, dark red), measured by flow cytometry. Mice per group for DCVAC OT-I-L, DMSO/Tg/pBr: CtrlLN *n* = 13/11/11 and dLN *n* = 11/13/12; mice per group for DCVAC CTR, DMSO/Tg/pBr: CtrlLN *n* = 5/5/6 and dLN *n* = 7/5/6, from 2 independent cohorts. (E) OT-I proliferation in the dLN, measured as decreased CFSE GeoMFI by FC. Lower CFSE GeoMFI indicates greater proliferation-induced CFSE dilution. Representative CFSE profiles are shown on the right. DMSO/Tg/pBr for DCVAC CTR and DCVAC OT-I-L are *n* = 11/13/12 and 8/5/6 mice, from 2 independent cohorts. Bars are means + SEM. For D–E, data were normalized within each experimental day (see “Statistics” in Materials and methods). Significant *p*-values were calculated using two-way ANOVA with Dunnett’s post-hoc test in A, C, and D, and one-way ANOVA with Dunnett’s post-hoc test in B and E. Exact *p*-values are shown above bars when significant, except in D, where all CtrlLN conditions were significantly different from their dLN counterparts, but *p*-values are not shown for clarity. # indicates significance compared with all conditions, except between TX controls in A.

The impact of SOCE modulators on DC viability and migration was next examined, as these are crucial factors for efficient DC-mediated T cell responses *in vivo.* ^6^ At the optimal concentrations for boosting T cell responses, neither compound induced significant cytotoxicity in LPS-matured BMDCs nor impaired their migration towards a gradient of CCL21 (Figure 2A, B). In a competitive co-transfer assay, CFSE-labeled DMSO- or SOCE modulator-treated LPS-matured CD45.1 BMDCs, loaded or not with OT-I-L, were mixed 1:1 with matched untreated, unlabeled CD45.1 BMDCs and injected into the footpad of CD45.2 recipient mice. CFSE? DMSO-, Tg-, and pBr-treated BMDCs were detected in the draining popliteal lymph node 24 h after injection, with no significant differences between SOCE modulator-treated and DMSO-vehicle-treated CFSE? BMDCs (Figure 2C, gating in Figure S2A). The OVAb format was excluded from these experiments as un-ingested OVAb could not be reliably separated from the BMDCs. Finally, in an *in vivo* BMDC/OT-I T cell stimulation assay, both compounds induced small but significant improvements in OT-I CD8^+^ T cell proliferation (Figure 2D–E, gating in Figure S2B). Whereas a significant antigen-specific increase in the total number of OT-I T cells in the dLN was only observed with pBr treatment (Figure 2D), CD25^+^CD69^+^was unchanged in antigen-loaded DCs (Figure S2C). Together, these findings indicate that SOCE modulators do not impair DC viability or grossly impair migration within 24 h and retain their potential to enhance DC-based vaccination *in vivo*.

### SOCE modulators enhance the efficacy of anti-melanoma DC-vaccines

**Figure 3.**
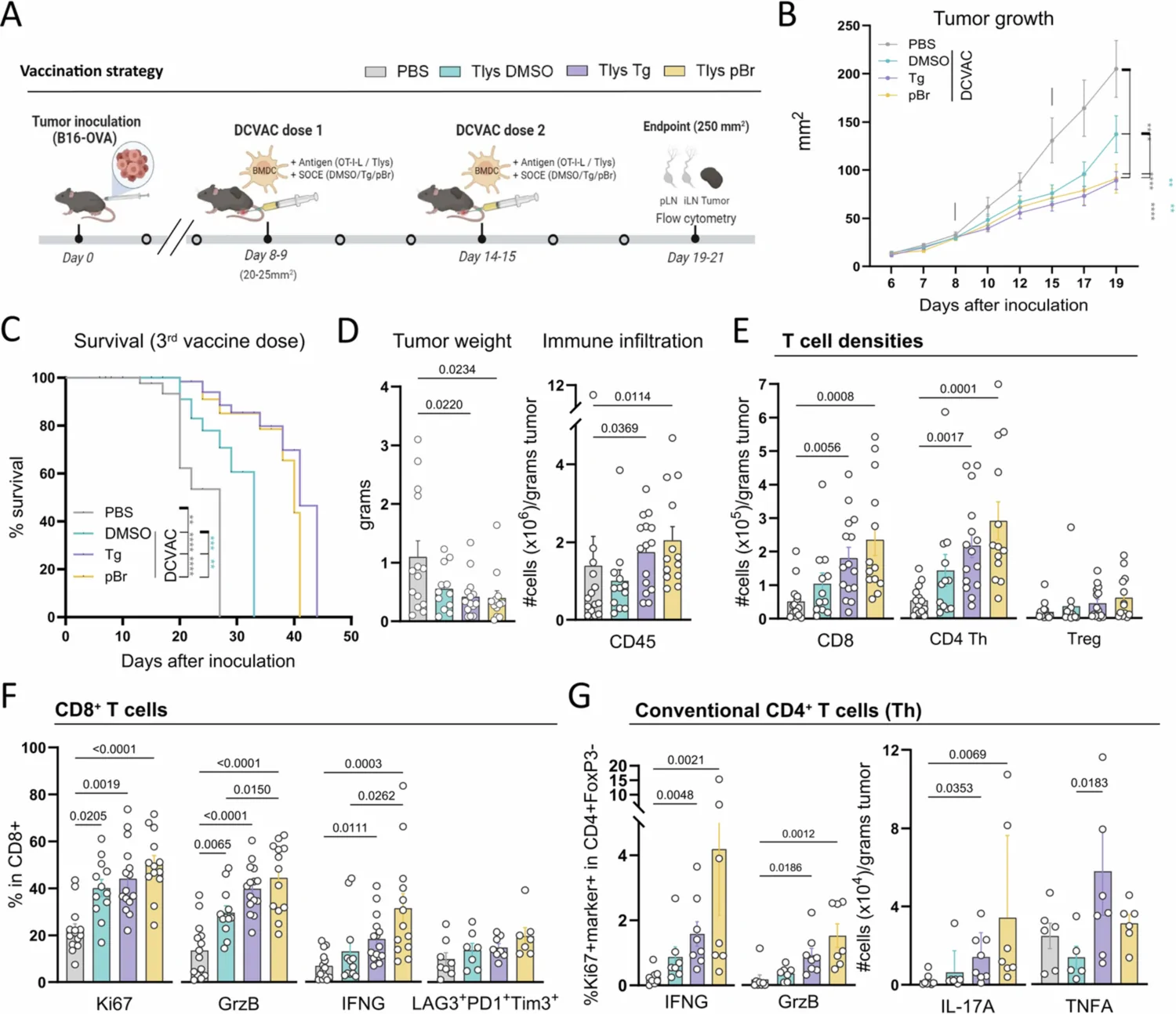
Impact of SOCE modulators on the efficacy of anti-melanoma DC-vaccines loaded with tumor lysate. WT C57BL/6J mice were inoculated with 0.5 x 10^6^ B16-OVA melanoma tumor cells, and tumor growth was monitored blindly every 2-3 d. Mice were vaccinated via the footpad twice (or 3 times in C) with BMDC vaccines (DCVAC). LPS-matured BMDCs were loaded with B16-OVA tumor lysate (Tlys; see also Figure 4 and Figure S3B–M), OT-I-L peptide (Figure S4), or no antigen (Figure S3A), and treated with Tg (purple), pBr (yellow), DMSO-vehicle control (cyan), or left unvaccinated (PBS, gray). All vaccination conditions were included within the same experimental cohorts. Tumors and draining lymph nodes (popliteal, pLN, and inguinal, iLN) were collected from a subset of mice and analyzed by flow cytometry (FC) at the endpoint. (A) Scheme of vaccination strategy used for A–B and D–G. For the survival experiment in C, a third vaccine dose was administered 8 d after the second dose. (B) Tumor growth curve (surface area, mm^2^). Arrows indicate vaccination (PBS/DMSO/Tg/pBr *n* = 20/22/21/22 mice, data pooled from 3 independent cohorts). (C) Survival curves (*n* = 10 mice per group). (D) Tumor weight at endpoint (grams; left), and leukocyte infiltration density (CD45?, right). (E) Tumor densities of T cell (TCR?) subsets: CD8^+^ T cells; conventional CD4^+^ T helper cells (Th, CD4^+^FOXP3^-^); regulatory T cells (Treg, CD4^+^ FOXP3?). (F) Frequency of functional markers on CD8? T cells: Ki67 (proliferation), granzyme B (GrzB; cytotoxicity), IFNG (activation), and terminal exhaustion markers (LAG3?PD1?TIM3?). (G) Frequency (left) and density (right) of functional and subset markers on CD4^+^ Th cells (Th1: IFNG; cytotoxicity: GrzB; Th17: IL-17A; activation: TNFA). Densities are expressed as cells per gram of tumor. Bars are means + SEM. Each dot in the bar graphs represents one biological replicate (mice per group in D–F: PBS/DMSO/Tg/pBr *n* = 14/12/15/13, from 2 independent experiments except for LAG3^+^PD1^+^TIM3^+^ CD8^+^ cells in F, where *n* = 8/7/8/7 from a single experiment. Mice per group in G: PBS/DMSO/Tg/pBr *n* = 8/7/8/7 for all except TNFA 6/5/7/6). Statistical analyses were performed using two-way ANOVA with Tukey’s post-hoc test in B, Kaplan–Meier with Log-rank Mantel–Cox test in C, and one-way ANOVA with Tukey’s post-hoc tests or Kruskal–Wallis non-parametric test with Dunn’s post-hoc test when normality assumptions were not met in D-G. For B and C, significant *p*-values are shown as *p < 0.05, **p < 0.01, ***p < 0.001, ****p < 0.0001. For D–G, exact *p*-values are indicated when significant.

Next, the potential of SOCE modulators in improving the performance of DC-based immunotherapy in a melanoma mouse model was investigated. When tumors established by intradermal injection of B16-OVA melanoma cells reached an average surface of 20–25 mm^2^, the mice were vaccinated with LPS-matured BMDCs loaded with either Tlys (Figure 3, Figure S3) or OT-I-L (Figure S4) and treated with SOCE-modulating compounds or DMSO vehicle control. Unvaccinated (PBS) mice were also used as controls. Vaccinated mice received a second dose after 6–7 d in all experiments and a third dose (8 d after the second) during survival measurements (Figure 3A–C, Figure S3, 4). At the endpoint, injection-draining popliteal, tumor-draining inguinal lymph nodes (pLN and iLN, respectively), and tumors were collected for flow cytometry analysis in a subset of animals. Significant improvement in tumor control was observed in mice receiving either Tg or pBr-treated BMDCs loaded with Tlys (Figure 3A, B). With the third dose, median overall survival was improved by 52% and 48% by Tg and pBr, respectively, whereas DMSO controls improved survival by only 22% (Figure 3C). Vaccination with BMDCs in the absence of antigens showed no effect or worsened tumor growth, highlighting the importance of antigen loading for DC vaccine efficacy (Figure S3A). While all vaccination conditions were evaluated within the same experimental cohorts, some variability was observed in the antigen-free control groups across experiments. Notably, the enhancing effect of SOCE modulation was consistently observed in antigen-loaded DC vaccination conditions, and conclusions are therefore focused on these comparisons. Overall, similar, though milder, results or trends were observed with the OT-I-L DC vaccines, where tumor growth was only inhibited in the pBr group compared to DMSO (Figure S4). With the Tlys DC vaccine, tumor weight was significantly decreased in the SOCE modulator but not in the DMSO group, while the infiltration density of CD45^+^ leukocytes was increased compared to PBS controls (Figure 3D, gating strategies in Figure S5). This included a lower percentage of proliferating tumor or tumor-associated (Ki67^+^CD45^-^) cells only in the Tg condition (Figure S3B). From within the leukocyte population, further analysis of the lymphoid and myeloid compartments was then performed.

In the T cell compartment, both SOCE modulators, but not DMSO, increased the tumor densities of both CD8^+^ and CD4^+^ conventional T helper (Th) populations, compared to unvaccinated controls (Figure 3E). In contrast, Treg density remained unchanged (Figure 3E). In lymph nodes, only minor changes were observed, including a slight increase in the Th/Treg ratio in the pBr group (Figure S3C–E). DC vaccination increased proliferation and GrzB expression in tumor-infiltrating CD8^+^ T cells (Figure 3F), and increased IFNG^+^ and GrzB^+^ CD8^+^ T cells were also detected in the pLN (Figure S3F). Importantly, SOCE modulators increased IFNG^+^ CD8^+^ T cells in the tumor compared to unvaccinated controls, and pBr treatment further enhanced both IFNG and GrzB expression in CD8^+^ T cells compared to DMSO treatment (Figure 3F). pBr also particularly increased IFNG^+^GrzB^+^ double-positive cells in tumors and IFNG^+^ and TNFA^+^ cells in the iLN (Figure 3F, Figure S3F, G). Moreover, both Tg and pBr enhanced TNFA^+^ CD8^+^ T cells in tumors compared to unvaccinated controls (Figure S3G), whereas terminal exhaustion markers were unchanged (Figure 3F). Within the Th cell compartment, DC vaccination generally increased proliferating IFNG^+^ and GrzB^+^ Th cells in the pLN, and increased proliferating cells in tumors (Figure S3H–J). Although global Th polarization phenotypes were largely unchanged, both Tg and pBr increased proliferating IFNG^+^ and GrzB^+^ Th cells in tumors (Figure 3G, Figure S3J). Tg and pBr also increased the tumor density of IL-17A^+^ and TNFA^+^ populations, and pBr increased TNFA^+^ Th cells in the iLN (Figure 3G, Figure S3H–J).

**Figure 4.**
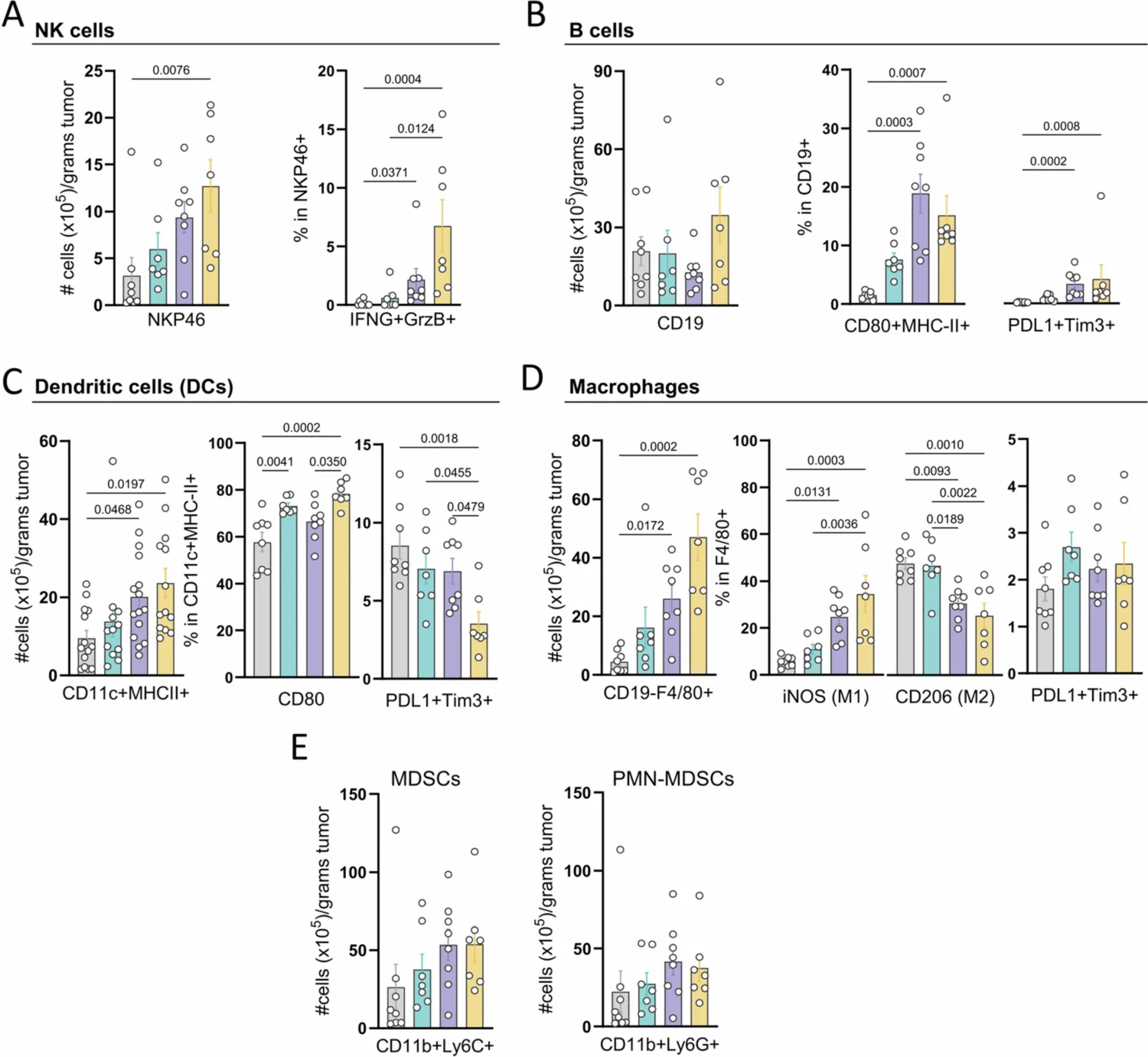
Impact of SOCE modulators on tumor-infiltrating immune cells after treatment with anti-melanoma DC-vaccines loaded with tumor lysate (see also Figure 3). (A) NK cell tumor infiltration, shown as density of (NKP46?) cells, and functional characteristics, activation (IFNG^+^); cytotoxicity (GrzB^+^). (B) B cell tumor infiltration, shown as density of (CD19?) cells, frequencies of activated (CD80?MHC-II?) B cells, and frequency of B cells co-expressing co-inhibitory markers PDL1 and TIM3. (C) DC infiltration, shown as density of (CD11c?MHC-II?CD19^-^F4/80^-^) cells, frequency of DCs expressing the activation marker CD80, frequency of DCs co-expressing the co-inhibitory markers PDL1 and TIM3. (D) Macrophage infiltration, shown as density of (F4/80?CD19^-^) cells, M1- and M2-associated phenotypes (iNOS? and CD206?, respectively), and frequency of macrophages co-expressing co-inhibitory markers PDL1 and TIM3. (E) Myeloid-derived suppressor cell infiltration, shown as monocytic MDSCs (F4/80^-^CD19^-^CD11b?Ly6C?), and polymorphonuclear MDSCs (PMN-MDSCs: F4/80^-^CD19^-^CD11b?Ly6G?). Bars are means ± SEM. Each dot in the bar graphs represents a biological replicate. Mice per group in A–B and D–E: PBS/DMSO/Tg/pBr *n* = 8/7/8/7. Mice per group in C: CD11c^+^ MHCII^+^ PBS/DMSO/Tg/pBr *n* = 14/12/15/13, from 2 independent experiments, CD80 and PDL1^+^ Tim3^+^ *n* = 8/7/8/7. Significant *p*-values were calculated using one-way ANOVA with Tukey’s post-hoc tests or Kruskal–Wallis non-parametric test with Dunn’s post-hoc test when normality assumptions were not met. Exact *p*-values are indicated when significant.

NK and B cells were also examined in a subset of tumors. NK cell recruitment was increased in the pBr group, and both Tg and pBr induced a more activated cytotoxic NK phenotype compared to controls, with a further increase in the pBr group compared to DMSO (Figure 4A). B cell recruitment was similar across all groups, but both SOCE modulators increased activation and co-inhibitory ligand expression (Figure 4B).

Myeloid cells were analyzed in tumors, with DCs also examined in lymph nodes (Figure 4C–E; Figure S3K–O). Although vaccine-derived CD45.1 BMDCs were not detected at this time point, the tumor density of endogenous DCs was increased by both SOCE modulators, but not DMSO, compared to unvaccinated controls (Figure 4C). In lymph nodes, pBr increased total and proliferating DCs in iLN, and DC proliferation increased across all vaccinated groups in pLN (Figure S3K). Tumor DC subset frequencies were unchanged, but CD80? DCs increased in the DMSO and pBr groups, while PDL1?TIM3? DCs decreased in the pBr group (Figure 4C, Figure S3L). Marker-intensity analysis suggested that this latter decrease was associated mainly with reduced TIM3 intensity in cDC subsets (Figure S3M, N). Macrophage tumor density was also increased by both SOCE modulators, with higher iNOS and lower CD206 expression (M1 and M2 associated-markers, respectively), whereas the tumor density of monocyte- or polymorphonuclear cell- derived suppressor cells (MDSCs or PMN-MDSCs, respectively) was unchanged (Figure 4D, E).

Overall, treating DCs with optimized SOCE modulators during antigen loading increased their ability to activate CD4^+^ and CD8^+^ T cells *in vivo* and promoted a broadly orchestrated immune response involving NK cells, B cells, and macrophages. Despite stronger immune responses with pBr, both compounds slowed tumor growth and increased survival to a similar extent, supporting their potential to improve DC-based immunotherapy designs.

### SOCE modulators induce distinct calcium signals and effects on antigen retention

To probe the mechanisms underlying the differences between Tg and pBr, intracellular calcium signals were assessed in LPS-matured BMDCs using the calcium indicator Fluo-8. Interestingly, in calcium-containing media, the two compounds produced similar global calcium signals at their optimal concentrations and were similarly sensitive to Orai and IP3R inhibition via GSK-7975A^23^ and Xestospongin C,^24^ respectively (Figure S6A–C). However, in calcium-free media, ER calcium release and calcium influx after extracellular calcium re-addition displayed measurable differences: pBr at 5 µM induced lower levels of ER calcium release, whereas calcium influx was much more strongly induced by Tg than by pBr at both concentrations (Figure S6D, E). These data suggest that differences in the rate of calcium influx or clearance may underlie the differences in the biological activities of these compounds.

To gain further insight into the mechanisms through which SOCE modulators affect DC function, the three essential signals provided by BMDCs during T cell activation^2^ were next examined. SOCE modulators did not increase surface or intracellular levels of peptide-MHC-I complex (MHC-I-*p*) detected with anti-H2Kb-SIINFEKL antibody across different antigen formats in LPS-matured BMDCs (Figure S7A–C). In contrast, quantification of intact antigens by anti-OVA immunofluorescence revealed significant differences in cells exposed to Tg but not pBr, indicating that Tg, but not pBr, may favor antigen persistence^25^ (Figure S7D). Thus, whether increased persistence could lead to improved antigen transfer, an important mechanism contributing to DC vaccine-mediated T cell priming,^26^ was assessed. However, although robust antigen transfer could be detected in co-cultures of OVA-647-loaded and antigen-naïve BMDCs, SOCE modulator stimulation did not have an impact (Figure S7E, F, gating strategy in Figure S8A–H). Similarly, surface levels of CD86, MHC-II, and 24 h supernatant levels of IL-12 were unchanged (Figure S7G–I). Thus, SOCE modulators had only limited effects on antigen presentation, transfer, and conventional activation markers.

### SOCE modulators boost human DC-driven T cell responses

**Figure 5.**
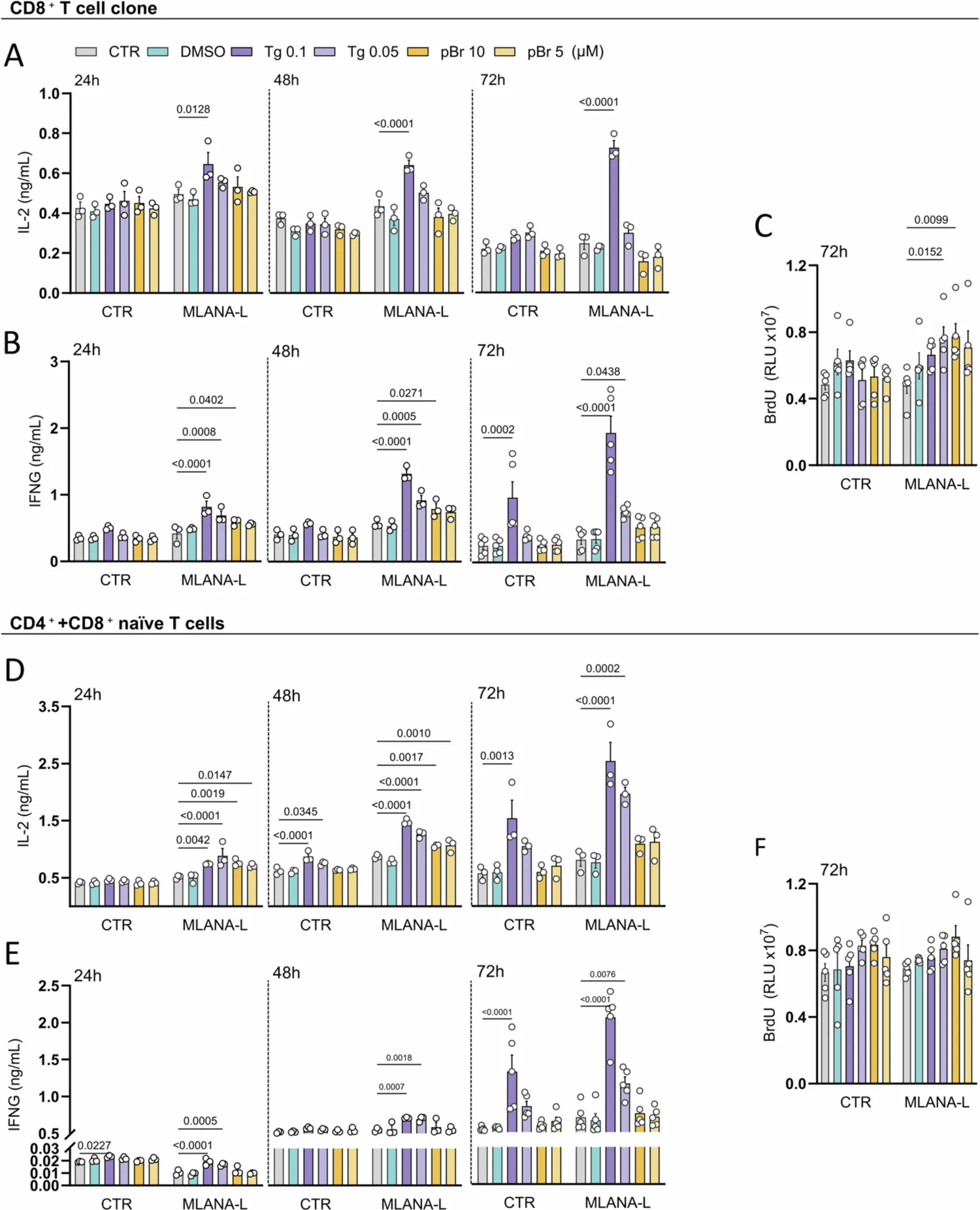
SOCE modulators boost human DC-mediated T cell responses. MoDCs matured with 5 ng/mL IL-1ß, 15 ng/mL IL-6, and 20 ng/mL TNFA were pulsed with 10 µM MLANA-L peptide or left unpulsed (CTR) and treated with SOCE modulators Tg (0.05–0.1 µM, light and dark purple) and pBr (5–10 µM, light and dark yellow), DMSO vehicle control (cyan), or no compound (CTR, gray). (A–C) MoDCs from 3 independent donors were co-cultured with a Melan-A-specific CD8? effector T cell clone (HD358). For BrdU assays in C and supernatant IFNG at 72 h in B, MoDCs from 2 additional donors were tested with clone 5B3. (A) Supernatant IL-2 and (B) IFNG levels were measured by ELISA at 24, 48, and 72 h using duplicate or triplicate wells. (C) Proliferation was measured at 72 h by BrdU incorporation using triplicate wells. (D–F) MoDCs from 3 independent donors were co-cultured with naïve allogeneic CD8? and CD4? T cells from a single donor at a 1:1:1 ratio. For supernatant IFNG at 72 h in (E) and BrdU assays in (F), MoDCs from 2 additional donors were tested together with T cells from a second allogeneic donor. (D) IL-2 and (E) IFNG levels were measured by ELISA at 24, 48, and 72 h using duplicate wells. (F) Proliferation was measured at 72 h by BrdU incorporation using triplicate wells. Bars are means + SEM. Each dot represents one independent MoDC donor, with technical replicate wells averaged before analysis. Data were normalized by experimental day to correct for inter-experimental variability in baseline signal intensity (see Materials and Methods). Significant *p*-values were calculated using two-way ANOVA with Dunnett’s post-hoc test, and exact values are shown above bars when significant.

To further explore the translational potential of SOCE modulators, MoDCs from HLA-A2^+^ healthy donors were matured, pulsed or not (CTR) with a long Melan-A peptide (MLANA-L)^27^ and treated with SOCE modulators as above. No toxicity of this 4 h SOCE modulator treatment was detected by supernatant lactate dehydrogenase release after overnight culture (Figure S9A). MoDCs were then washed and co-cultured with Melan-A-specific CD8^+^ T cell clones or with naïve allogeneic HLA-A2^+^ CD8^+^ and CD4^+^ T cells, since robust human naïve CD8^+^ T cell priming often requires CD4^+^ T-cell help.^28^ IL-2 and IFNG were measured in co-culture supernatants at 24, 48, and 72 h, and proliferation was measured at 72 h. In Melan-A-specific CD8^+^ T cell clone co-cultures, 0.1 µM Tg significantly increased IL-2 and IFNG production at all time points, although a non-antigen-specific effect on IFNG was observed at 72 h (Figure 5A, B). pBr did not affect IL-2 but significantly increased IFNG at 24 and 48 h, and both Tg and pBr induced a small increase in antigen-specific proliferation at 72 h (Figure 5A–C). In naïve allogeneic co-cultures, Tg significantly boosted antigen-specific IL-2 and IFNG production at all time points, although non-antigen-specific activation was also observed at 72 h (Figure 5D, E). pBr significantly increased IL-2 production at 24 and 48 h but did not significantly increase IFNG or proliferation (Figure 5D–F).

To further define the cellular source and antigen dependence of these responses, co-cultures between autologous HLA-A2^+^ MoDCs and CellTrace-Violet (CTV) labeled total T cells were next examined. To increase the probability of detecting antigen-dependent responses, peptide pools containing either long or HLA-A2-compatible minimal peptides from three melanoma-associated antigens, Melan-A, NY-ESO, and gp100, were compared with no-antigen controls. In addition, to test whether broad calcium mobilization was sufficient to phenocopy the effects of Tg, MoDCs were treated with ionomycin in parallel with SOCE modulators. After 48 h of co-culture, cells were analyzed by flow cytometry for CTV dilution as a measure of proliferation and intracellular IL-2 and IFNG expression. Interestingly, antigen-dependent proliferation of both CD4^+^ and CD8^+^ T cells could only be detected in the presence of Tg and both short or long peptide pools, whereas pBr induced significant proliferation only in CD4? T cells in the presence of short peptides (Figure S9B, gating strategy Figure S10A–Q). Moreover, Tg increased the percentage of IL-2^+^, IFNG^+^, and IL-2^+^IFNG^+^ CD4^+^ T cells and IFNG^+^ CD8^+^ T cells across antigen conditions. In contrast, significant antigen-dependent increases were most evident for IL-2? and IL-2?IFNG? CD8? T cells (Figure S9C–E). Tg also increased cytokine-positive MoDCs mainly in the absence of antigen, whereas ionomycin did not significantly increase cytokine-positive MoDCs or T cells under these conditions (Figure S9C–E). Together, these data confirm that SOCE modulators, particularly Tg, can improve human T-cell responses to antigen-loaded MoDCs and suggest that Tg-mediated boosting cannot be readily reproduced by ionomycin treatment under these conditions.

### SOCE modulators rewire DC transcriptional and secretory programs associated with enhanced T cell stimulation

**Figure 6.**
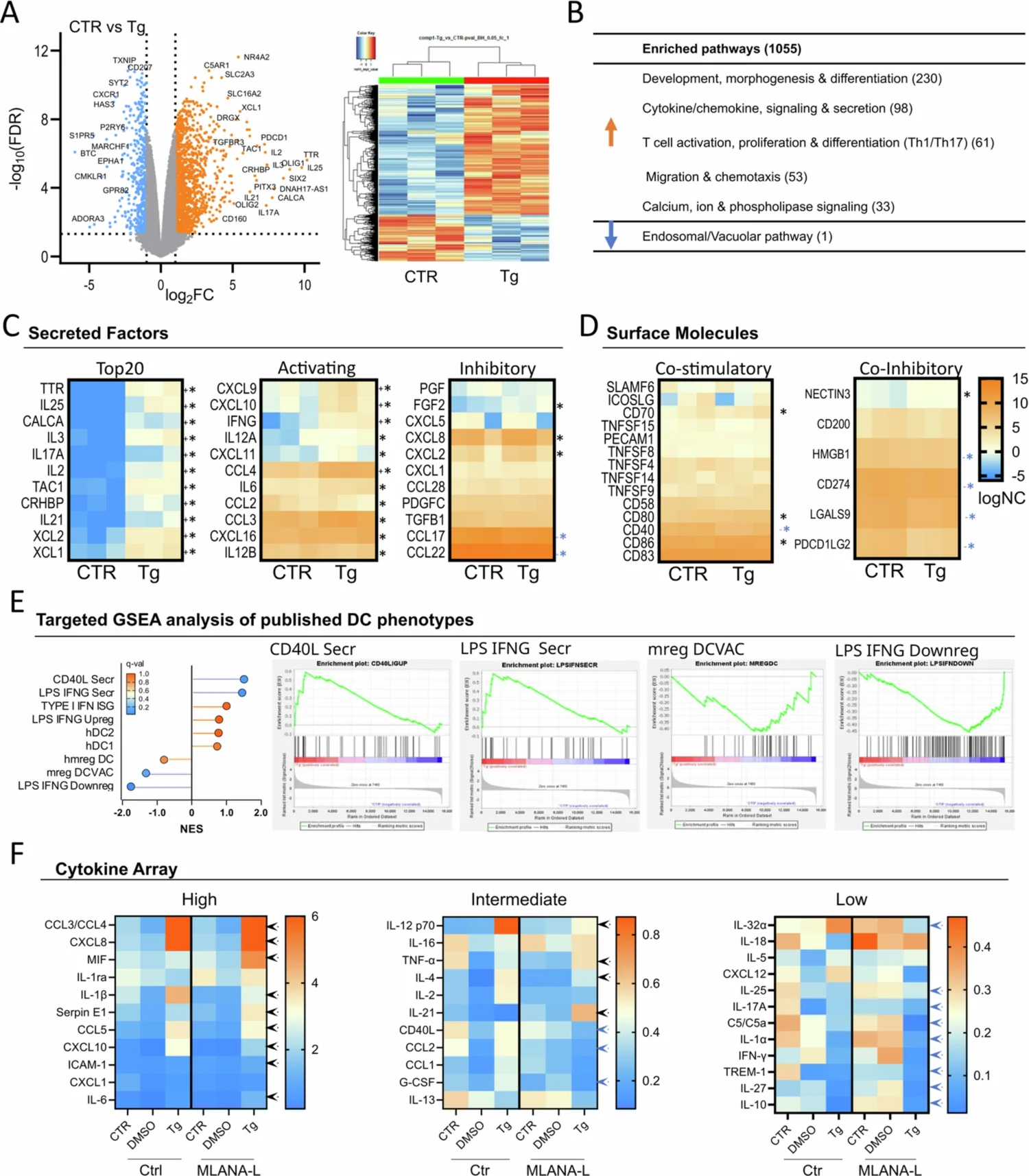
Broad reprogramming of human MoDCs by Tg. Bulk RNA sequencing analysis of MoDCs treated with 0.1 µM Tg or untreated (CTR) (*n* = 3 donors per condition, see also Supplementary Data S1). (A) Volcano plot (log_2_ fold-change, FC vs -log_10_ False Discovery Rate, FDR) highlighting differentially upregulated (1408 genes, orange) and downregulated (507 genes, blue) genes after Tg treatment (FC = 2, FDR < 0.05, gene symbols of the most significantly differentially expressed genes are shown, see also Supplementary Data S1). A clustered heatmap is shown on the right, where each column within treatment condition represents an independent donor. The color scale represents expression level (blue to orange = low to high). (B) Summary of 1055 enriched pathways identified through overrepresentation analysis of Gene Ontology Biological Process, Molecular Function, and KEGG pathways, together with unsupervised gene set enrichment analysis (fGSEA) of WikiPathways, Reactome, and Hallmark MSigDB pathway databases. The table shows the number of pathways grouped into functional categories based on word frequency analysis, with the number of enriched pathways containing the keyword shown in brackets (see also Supplementary Data S1). (C) Heatmaps showing differential expression of secreted factors after Tg treatment. Left: secreted factors present within the top 20 upregulated genes after Tg treatment, ranked by fold-change. Middle: factors with a known activating effect on T cells. Right: factors with a known inhibitory effect on T cells. (D) Heatmaps showing differential expression of co-stimulatory (left) and co-inhibitory (right) surface molecules (based on references 3, 30). (C, D) NC: Normalized Counts. *: Significantly differentially expressed. + *: = 2-fold upregulation. -*(blue): = 1-fold downregulation. Each column within treatment condition represents an independent donor. (E) Targeted GSEA examining the correspondence between Tg-induced gene expression changes and previously published DC phenotype signatures. Left panel: Lollipop plot showing normalized enrichment scores (NES) for various DC phenotypes, with color scale representing q-values (blue to red). Right panels: Enrichment score plots for the significantly differentially expressed phenotypes. CD40L/LPS IFNG Secr: Group of factors secreted upon stimulation of DCs with CD40L (CD40L Secr) or LPS and IFNG (LPS IFNG Secr); TYPE I IFN ISG: stimulatory DC vaccine phenotype correlating with positive clinical performance; LPS IFNG Upreg/Downreg: Group of genes upregulated (Upreg) or downregulated (Downreg) upon stimulation of DCs with LPS and IFNG; hDC2,hDC1: Group of genes associated with human cDC2, or cDC1 phenotypes; hmregDC: Group of genes associated with regulatory (suppressive) DC phenotype correlating with negative clinical performance. (F) Cytokine array screening of supernatants collected after 4 h treatment and overnight culture from matured MoDC cultures left untreated (CTR) or treated with DMSO or Tg, in the presence or absence of MLANA-L antigen, pooled from 3 different donors (see also Supplementary Data S2). For visualization, RLU values were normalized to the mean signal across all cytokine spots from the arrays analyzed in the same image. Panels are divided into high-, intermediate-, and low-expression sections for clarity. Black and blue arrows indicate cytokines upregulated or downregulated by Tg, respectively, compared with either CTR or DMSO conditions in the presence of MLANA-L antigen.

Since modulation of DC SOCE had only minimal effects on antigen presentation and conventional activation markers (Figure S7), we turned to an unbiased transcriptomic approach to capture more deeply the impact of these compounds. Bulk RNA sequencing was performed on human MoDCs, as we reasoned that profiling the human system would yield the most relevant insights for translational applications. Tg treatment induced a significant differential expression of 1915 genes (1408 upregulated, 507 downregulated, out of 16,509 identified genes), indicating that Tg induced extensive cellular reprogramming (Figure 6A, B, Data S1). Overrepresentation analysis (ORA) of gene ontology (GO), Biological Process (BP), Molecular Function (MF), and Kyoto Encyclopedia of Genes and Genomes (KEGG) pathways, as well as unsupervised gene set enrichment analyses (fGSEA) probing the WikiPathways, Reactome, and Hallmark MSigDB databases, identified significant differences in 1,055 pathways. To reduce dimensionality and identify dominant biological themes, keyword searches were performed using word frequency analysis, followed by manual curation of the pathways into functionally coherent categories. Interestingly, the pathways involved in development, morphogenesis, and differentiation (not specific to T cells, including myeloid cells) were the most highly represented, with 230 pathways fitting into this category. However, cytokine and chemokine secretion and signaling (98 pathways) were the most prominent themes detected across all six pathway analysis algorithms (ORA and fGSEA). In addition, T cell activation, proliferation, and differentiation (including Th1 and Th17) (61 pathways); migration and chemotaxis (53 pathways); and calcium-, ion-, and phospholipase-related signaling (33 pathways) also emerged as prominent themes (Figure 6B, Figure S11A, B, Data S1). In contrast, the endosomal/vacuolar pathway was the only significantly downregulated pathway identified, whereas no significant changes in pathways related to antigen processing or presentation were detected (Figure 6B, Data S1). Scrutiny of the top 20 significantly upregulated genes (sorted by the highest fold-change) revealed that 11/20 were secreted factors associated with both positive and negative effects on inflammation (Figure 6C, Top20). However, a closer examination of selected secreted factors shown to promote (Figure 6C, Activating) or inhibit (Figure 6C, Inhibitory) T cell function in tumors^29^ suggested a greater trend towards the secretion of activating factors rather than that of inhibitory factors. Differential expression of selected co-stimulatory and co-inhibitory surface molecules^3^ ^,^ ^30^ did not indicate major changes in co-stimulation, but still suggested a trend towards slightly reduced co-inhibition (Figure 6D). Finally, targeted gene set enrichment analysis (GSEA) was conducted to examine the enrichment of published gene sets defining cDC1, cDC2, or inhibitory (mreg) DC phenotypes, as well as gene sets associated with mature or activated states.^31–33^ Interestingly, two activation phenotypes (representing upregulated secretion upon CD40L and LPS/IFNG stimulation^33^) were significantly enriched, whereas two inhibitory phenotypes (mregDC associated with poor DC vaccine performance^31^ and genes downregulated upon LPS/IFNG stimulation) were significantly suppressed (Figure 6E). A leading-edge analysis of the GSEA results was also performed to identify the genes within the gene sets that drove the enrichment scores. This analysis showed that genes driving the activation phenotypes included CXCL9, IL12A, CCL4, CXCL10, CXCL11, and IL6, whereas genes associated with the suppression of the inhibitory phenotypes included T cell cytokines and ligands CCL22, PD-L1 (CD274), PD-L2 (PDCD1LG2), LGAL9 (galectin-9, a TIM3 ligand), and CCL19. In contrast, treatment with pBr did not induce significant changes in gene expression using a cut-off of log fold-change (logFC) > 2, where significant changes showing logFC > 1 consisted of only seven upregulated genes, four of which were secreted factors of the metallothionein family (Figure S11C, D, Data S1). These observations are consistent with the milder effect of pBr in human cells than in murine cells.

To verify that transcriptional changes indeed translate into alterations in the DC secretome, a 36-analyte array analysis was performed. MoDCs were left untreated or exposed to DMSO or Tg for 4 h in the presence or absence of MLANA-L antigen, then washed and cultured overnight before supernatant collection. Tg markedly remodeled the MoDC secretome under both antigen conditions, with the strongest differences observed relative to DMSO-treated controls (Figure 6F, Figure S12A, B, Data S2). Across conditions, Tg consistently increased CCL3, CCL4, and CCL5, together with ICAM-1 and TNFA, as well as additional mediators that overlapped with the transcriptional response, including CXCL10, CXCL8, IL-6, IL-12, IL-2, and IL-21. In contrast, IL-17A, IL-25, and IFNG were reduced, while counter-regulatory mediators such as IL-1RA and Serpin-E1 were increased, indicating that Tg induced a mixed but strongly remodeled secretory program. Since these data pointed to secretory reprogramming as a candidate mechanism, we also examined whether related changes could be detected in murine BMDCs across the antigen formats and SOCE modulators. Detecting a similar remodeling of soluble mediators in this setting would strengthen the idea that secretory reprogramming contributes to the enhanced T cell responses observed across systems. BMDCs were analyzed using a 40-analyte array after a shorter 4 h collection window, in the presence of Tg or pBr, and either OT-I-L or OVAb antigens were examined, allowing comparison of early secretory responses across antigen formats and SOCE modulators (Figure S12C–E, Data S2). Although the response varied with antigen format, both SOCE modulators altered soluble mediator release. With OT-I-L stimulation, TIMP-1, CXCL1, and soluble ICAM-1 were increased by both Tg and pBr relative to DMSO, with CXCL1 and ICAM-1 also increased by Tg in MLANA-L-loaded human MoDCs. Interestingly, with OVAb, stronger responses to Tg and pBr were observed with a broad increase in cytokines and chemokines relative to both PBS and DMSO controls (Figure S12C–E, Data S2). Here, several mediators common to the human MoDC response to Tg included CCL3, IL-12, IL-1RA, CXCL10, and soluble ICAM-1. Additional factors increased by both compounds included CXCL11, CCL11/eotaxin, and C5/C5a, whereas IL-1ß was downregulated. Together, these transcriptome and secretome analyses strongly suggest that SOCE modulators potentiate T cell responses to antigen-loaded DCs in both mice and humans, not by increasing antigen presentation, but by reprogramming inflammatory, chemokine, and regulatory soluble mediator secretion. This provides a mechanistic framework for how SOCE-modulated DCs can potentiate antigen-dependent T cell responses across human and murine systems.

## Discussion

Effective T cell stimulation requires several calcium-dependent DC functions, including antigen uptake, processing, and loading on MHC molecules (signal 1); chemokine-guided migration towards T cells; co-stimulatory molecules expression (signal 2); and stimulatory cytokines secretion (signal 3). Here, pharmacological manipulation of SOCE, the primary calcium signaling pathway in DCs, led to several insights into how this pathway shapes DC function. First, although Tg increased the intracellular levels of intact antigens, antigen presentation via MHC-I was not enhanced, contrary to our initial hypothesis but in line with previously observed dual enhancing and inhibitory effects on antigen processing.^15^ Indeed, our RNA-seq analysis revealed a similarly conflicting pattern in antigen processing. With pBr, MHC-I-*p* complexes were depleted, even though *in vivo* pBr improved peptide DC vaccine performance compared to DMSO. Although we cannot exclude that exosomal secretion of MHC-I-*p* complexes might occur and contribute in this context,^9^ changes in signal 1 are likely not the driving mechanism. Similarly, our data agree with previous reports^13^ on the minimal impact of SOCE on co-stimulation. However, both our RNA-seq and *in vivo* analyses suggest that an altered co-inhibitory marker pattern, potentially involving the PD-L1, PD-L2, or TIM3 axes, may contribute to an indirect strengthening of signal 2.

In addition, our cytokine array and RNA-seq analyses suggest that a global reprogramming of secreted signals may be the most important driving factor. Indeed, short-term calcium-induced protein-level changes in secretion may help explain how pBr could promote T cell activation. Notably, one unexpected inference drawn from the cytokine array analysis of soluble peptides versus solid particles is that SOCE modulators induce a broader secretory response in the context of solid particle antigens. Although phagocytosis has long been known to boost DC-mediated T cell responses, the underlying mechanisms are not entirely understood and have tentatively been linked to improved antigen processing (signal 1).^20^ ^,^ ^34^ ^,^ ^35^ Our data indicate that a previously unrecognized signal 3 cytokine component may also contribute, although further verification of this hypothesis is required. By the same reasoning, one may speculate that solid debris found in uncleared Tlys may not only provide a greater breadth of antigens, but also stimulate secretory reprogramming; together, these effects may thus contribute to the ability of Tlys to potentiate anti-tumor responses.^36^ Another surprising finding was that, despite generally weaker effects *in vitro,* pBr produced stronger *in vivo* immunostimulatory effects across almost all of the immune cell populations examined. It was also more effective in stimulating the OT-I-L peptide DC vaccine. Whether this difference is due to stronger stimulation by pBr or whether it is due to the simultaneous induction by Tg of counterregulatory factors, for example, calcitonin gene-related peptide (CALCA),^37^ remains to be investigated. Moreover, although no gross defects in migration or toxicity were observed under the conditions tested with Tg, transcriptional pathways associated with the negative regulation of migration and cellular stress were significantly enriched by Tg. Thus, long-term deficits in migration or survival of Tg-treated DCs may reduce the full potential of Tg stimulation to promote DC function *in vivo.*

Overall, our study demonstrates that SOCE modulation induces complex cellular reprogramming that promotes a secretory phenotype and boosts the ability of DCs to orchestrate inflammatory immune responses. More broadly, our findings highlight SOCE as a targetable pathway within DCs to improve DC vaccine efficacy. Current efforts include strategies aimed at optimizing antigen source and loading, DC maturation, lymph-node migration, genetic engineering, and combination with other immunotherapies.^3^ ^,^ ^6^ ^,^ ^7^ Although the use of Tg as an immunostimulant in the context of ex vivo DC preparation has been suggested previously,^38^ ^,^ ^39^ in these studies, Tg-stimulated LAD2 mast cells, or their released products, were instead utilized to induce maturation in MoDCs. In contrast, our aim was to mimic natural phagocytic calcium signals in DCs, and our short-duration, low-dose pharmacological approach is distinct in that it boosts downstream DC–T-cell responses in already matured DC preparations. Finally, our findings suggest that transient pharmacological modulation of SOCE could be explored as a strategy to improve DC-dependent detection or expansion of tumor-reactive T cells, thereby complementing or informing immunotherapy designs based on the DC-T cell axis.

## Materials and methods

### Antibodies, dyes, and chemicals

Fc-Block anti-mouse/humanCD16/CD32 FcgRII-RIII (clone 93, #14-0161-86, Invitrogen), FITC anti-mouse CD11c (clone N418, #117305, Biolegend), APC anti-mouse CD11c (clone HL3, #550261, BD Biosciences), BUV737 anti-mouse CD11c (clone HL3, #612796, BD Biosciences), PE anti-mouse CD11c (clone N418, #117305, Biolegend), AF647 anti-mouse I-Ab, MHC-II (clone KH74, #115309, Biolegend), FITC anti-mouse I-Ab, MHC-II (clone KH74, #115305, Biolegend), PE anti-mouse CD86 (clone GL-1, #105007, Biolegend), PerCP/Cy5.5 anti-mouse CD80 (clone 16-10A1, #104722, Biolegend), APC anti-mouse CD80 (clone 16-10A1, #104713, Biolegend), PE-Cy7 anti-mouse CD40 (clone 44986, #124621, Biolegend), PE-Cy7 anti-mouse H-2Kb-SIINFEKL, MHC-I-*p* (clone 25-D1.16, #141607, Biolegend), Biotin anti-mouse H-2Kb-SIINFEKL, MHC-I-*p* (clone 25-D1.16, #13-5743-82, ThermoFisher), Anti-mouse H-2Kb-SIINFEKL, MHC-I-*p* (clone 25-D1.16, #17-5743-80, ThermoFisher), BV421 anti-mouse CD45.1 (clone A20, #110732, Biolegend), FITC anti-mouse TCR Vß5.1, 5.2 (clone MR9-4, #139513, Biolegend), PE-Cy7 anti-mouse CD8a (clone 53-6.7, #100722, Biolegend), APC anti-mouse CD69 (clone H1.2F3, #104514, Biolegend), BV605 anti-mouse CD25 (clone PC61, #563061, BD Biosciences), BV786 anti-mouse CD45 (clone 30-F11, #564225, BD Biosciences), BV650 anti-mouse CD3 (clone 17A2, #100229, Biolegend), BUV395 anti-mouse CD4 (clone RM4-5, #740208, BD Biosciences), PE-eFluor610 anti-mouse CD8a (clone 53-6.7, #61-0081-82, ThermoFisher), BV711 (SB702) anti-mouse CD11b (clone M1/70, #67-0112-82, ThermoFisher), BV510 anti-mouse Ly6C (clone HK1.4, #128033, Biolegend), BV605 anti-mouse Ly6G (clone 1A8, #127639, Biolegend), BV421 anti-mouse XCR1 (clone ZET, #148216, Biolegend), AF700 anti-mouse I-A/I-E, MHC-II (clone M5/114.15.2, #107622, Biolegend), PE anti-mouse/Rt FoxP3 (clone FJK-16s, #12-5773-82, ThermoFisher), PE-Cy7 anti-mouse IFNg (clone XMG1.2, #25-7311-82, ThermoFisher), PerCP-eF710 anti-mouse TNFa (clone MP6-XT22, #46-7321-82, ThermoFisher), FITC anti-mouse TNFa (clone MP6-XT22, #RM9011, ThermoFisher), AF647 anti-mouse/Hu Granzyme B (clone GB11, #515406, Biolegend), FITC anti-mouse/Rt Ki67 (clone SolA15, #11-5698-82, ThermoFisher), PerCP-eF710 anti-mouse Ki67 (clone SolA15, #46-5698-82, ThermoFisher), FITC anti-human HLA-A2 (clone BB7.2, #343303, Biolegend), BV421 anti-human HLA-DR (clone L243, #307635, Biolegend), BB515 anti-human CD11c (clone B-ly6, #564490, BD Biosciences), PerCP/Cy5.5 anti-human CD80 (clone 2D10, #305231, Biolegend), PE/Cy7 anti-human CD40 (clone 5C3, #334321, Biolegend), PE anti-human CD86 (clone BU63, #374205, Biolegend), APC anti-human CD14 (clone TuK4, #MHCD1405, ThermoFisher), PE-Cy7 Anti-human CD3 (clone UCHT1, #25-0038-41, ThermoFisher), BV570 anti-human CD3 (clone UCHT1, #300436, Biolegend), APC anti-human CD8 (clone SK1, #344721, Biolegend), BUV737 anti-human CD8 (clone SK1, #612755, BD Biosciences), APC anti-human CD4 (clone OKT4, #317415, Biolegend), APC-R700 anti-human CD4 (clone SK3, #566909, BD Biosciences), BV786 anti-human IFNG (clone 4S.B3, #563731, BD Biosciences), PE anti-human IL-2 (clone MQ1-17H12, #500307, Biolegend), Anti-Ovalbumin (egg white, anti-OVA) (#23744-5, PolySciences), AF488 goat anti-rabbit (GAR) (#A-11034, ThermoFisher), AF488 goat anti-mouse (GAM) (#A-11029, ThermoFisher), AF594 Streptavidin (#405240, Biolegend), carboxyfluorescein-succinimidyl ester (CFSE, #65-0850-84, ThermoFisher), propidium iodide (PI) (#P4864, Sigma), Hoechst 33342 (#H3570; ThermoFisher), DAPI (#A4099-0005, PanReac AppliChem), DRAQ5 (#65-0880-92, ThermoFisher), DRAQ7 (#D15106, ThermoFisher), Fixable viability dye eFluor 780 (APC-Cy7) (#65-0865-18, ThermoFisher), LIVE/DEAD Fixable Aqua Dead Cell Stain Kit (LDAqua) (#L34966, ThermoFisher), LIVE/DEAD Fixable Near IR (780) Dead Cell Stain Kit (LD-NIR) (#L34966, ThermoFisher), CellTrace Violet Cell Proliferation kit (CTV, #C34557, ThermoFisher), Ovalbumin Alexa Fluor 647 Conjugate (OVA-AF647, #O34784, ThermoFisher). Chemicals were purchased from Carl Roth AG, with the following exceptions: from Sigma/Merck: sulfinpyrazone (#S9509), pluronic F-127 (#P-2443), PIPES (#P6757), saponin (#47036), phorbol 12-myristate 13-acetate (PMA, #P1585), ionomycin (#I0634), 2-aminoethyl diphenylborinate (2-APB, #D9754), and cyclopiazonic acid (CPA, #C1530). Chemicals from other sources include: Xestospongin C (64950 Cayman Chemical), GSK-7975A (#AOB4124, Aobius), resazurin (#199303, Millipore), thapsigargin (Tg, #BML-PE180, Enzo Life Sciences), platelet activator factor (PAF, #2940, Tocris), paraformaldehyde (PFA #ALF043368-9M, AlfaAesar), or as specified below.

### Ethics statement – animal studies

All animal procedures were reviewed by the Commission cantonale pour les expériences sur les animaux and approved by the Service de la Consommation et des Affaires Vétérinaires of the Canton of Geneva, Switzerland. Animal ethic approval numbers (federal/cantonal) are 33360/GE55 (study period March 1st 2021–March 1st 2024), 35688/GE287 (study period April 24th 2023–April 9th 2024), replaced by the amendment 35688/GE287A/(study period April 10th 2024–May 24th 2026). All animal procedures were conducted in accordance with institutional guidelines and Swiss Federal animal welfare legislation. Experiments are reported in compliance with the ARRIVE guidelines. A completed ARRIVE checklist is provided as ARRIVE Checklist (pdf).

### Ethics statement – human materials

Human biological materials used in this study consisted of human peripheral blood mononuclear cells (PBMCs) isolated from buffy coats obtained from healthy blood donors through the Geneva University Hospitals Blood Transfusion Center. The buffy coats were supplied under a non-therapeutic research supply agreement, which guarantees donor anonymity to the research team. The research team had no access to donor-identifying information, clinical information, or any re-identification key. Melan-A/MART-1-specific CD8? T-cell clones were purchased from HemaCare/Cellero (Charles River) as fully anonymized donor-derived materials supplied under the vendor’s donor consent and IRB/ethical compliance framework. No direct interaction with human subjects occurred at our institution. The research adhered to the principles of the Declaration of Helsinki where applicable. In accordance with the Swiss Federal Act on Research involving Human Beings/Human Research Act (HRA; SR 810.30, see Art. 2 https://www.fedlex.admin.ch/eli/cc/2013/617/en), research involving anonymized human biological material does not require ethics committee approval. Accordingly, no ethics approval number is available for this part of the study.

### Animal studies

Wild-type (WT) CD45.2 C57BL/6J and WT CD45.1 C57BL/6J mice were purchased from Charles River Laboratories, France or Italy, or bred in-house at the animal facility of the University of Geneva (UNIGE). Mice shipped from Charles River were acclimatized for 1 week prior to inclusion in experiments. Ovalbumin peptide (OVA_257–264_)-specific TCR transgenic OT-I CD45.1 mice (derived from C57BL/6-Tg(TcraTcrb)1100Mjb/J mice, a kind gift from Prof. S. Becattini, UNIGE)^40^ were bred at the University of Geneva animal facility at the Faculty of Medicine. The genotype was verified by flow cytometry analysis of OT-I TCR Vß5.1, 5.2, and CD45.1 expression. Ovalbumin peptide (OVA_323-339_)-specific TCR transgenic OT-II mice (C57BL/6-Tg(TcraTcrb)425Cbn/Crl)^41^ were bred and handled at the INSERM U1151 animal facilities. OT-II mice were bred and maintained at INSERM U1151 (Paris, France). Cells derived from OT-II mice used in this study were obtained from surplus material generated under ongoing licensed experimental procedures at INSERM in accordance with French national regulations. No additional regulated animal procedures were performed at the University of Geneva. All experiments carried out in Geneva were conducted under the approvals GE55 and GE287A. All mice were bred and maintained under specific pathogen-free conditions, with environmental enrichment, and were used between 6 and 15 weeks of age. The experimental unit for all *in vivo* analyses was a single mouse (i.e., data points represent individual animals rather than cages or litters). For *in vitro* assays (e.g., BMDC co-cultures) and *in vivo* migration assay, both male and female mice were used. For *in vivo* T cell responses experiments, male recipients and male OT-I donors were used to match colony availability and donor/recipient compatibility. In pilot tumor-growth studies, male cohorts exhibited aggression (fighting/biting of tumors) that confounded measurements and increased ulceration; therefore, all tumor-growth and survival experiments were conducted in female mice to avoid aggression-related bias and welfare concerns. Animals were allocated to experimental groups within single-sex cohorts for each model (male-only T cell responses, female-only tumor studies); no formal randomization procedure was applied for these assays. Anesthesia was induced by intraperitoneal injection of 100 µL of 13 mg/kg xylazine (Rompun 2%, Bayer) and 65 mg/kg ketamine 10% (Vetoquinol). Euthanasia was performed by intraperitoneal injection of 100 µL of 150 mg/kg pentobarbital in 0.9% NaCl (Esconarkon; Streuli Pharma SA).

### Cell lines, primary, and primary-derived *ex vivo* cultured cells

All cell culture media and supplements were purchased from Gibco unless otherwise specified. Supplements included Fetal Bovine Serum (FBS, #10270106), 4 mM L-glutamine (L-glu, #25030), 1 mM sodium pyruvate (#11360039), 50 µM *ß*-mercaptoethanol (*ß*-ME,#31350010), 800 µg/mL geneticin (G418, #10131035), 1X MEM Non-essential amino acids (NEAA #11140035), 1% penicillin-streptomycin (pen-strep, #P0781, Sigma), HEPES (#P05-01100, PanBiotech), human serum (HS #H4522, Sigma), mouse GM-CSF (#315-03, Peprotech), human GM-CSF, and IL-4 (#300-03 and #200-04, Peprotech). All cells were maintained at 37°C/5% CO2. Cell cultures were tested for mycoplasma infection every 6 months, and data gathered from mycoplasma-infected cells were excluded.

#### Cell lines

JAWSII cells (#CRL-11904, ATCC)^42^ were maintained in alpha-MEM with ribonucleosides and deoxyribonucleosides (#22571), supplemented with 20% FBS, pen-strep, L-Glu, sodium pyruvate, and 5 ng/ml GM-CSF. B3Z CD8^+^ T cell hybridoma cells^43^ were maintained in RPMI 25 mM HEPES L-Glu (#52400025) supplemented with 10% FBS, pen-strep, sodium pyruvate, *ß*-ME, and G418 to maintain CD8 expression. B16-OVA melanoma cells^21^ were maintained in RPMI medium supplemented with 10% FBS, pen-strep, *ß*-ME, and 400 µg/mL G418 to maintain OVA expression.

#### Primary and primary-derived ex-vivo cultured murine cells

BMDCs were generated by culturing bone marrow cells isolated from femurs and tibias 6–15-week-old male and female mice in BMDC medium (DMEM 4.5 g/L glucose L-glu (#41965039), supplemented with 10% FBS, pen-strep, sodium pyruvate, *ß*-ME, and 40 ng/mL GM-CSF), refreshed every 3 d, in non-treated dishes. Non-adherent and semi-adherent cells were harvested and used between days 7 and 10 of culture, and verified to be > 80% CD11c^+^MHC-II^+^ by flow cytometry. Maturation and activation levels were evaluated for CD80/86 and CD40 expression. Primary naïve CD8^+^ OT-I T cells were obtained from OT-I mouse spleens using the MagniSort Mouse CD8 naïve T cell enrichment kit (#8804-6825-74, ThermoFisher). The cell purity was verified to be > 95% CD8a^+^ by flow cytometry.

#### Primary and primary-derived ex-vivo cultured human cells

PBMCs were isolated by Lymphoprep (#1114545, Axis-Shield) density gradient centrifugation after HLA-A2 haplotype verification by flow cytometry. Monocytes were isolated using the MagniSort Human CD14 positive selection kit (#8802-6834-74, ThermoFisher) and further differentiated into dendritic cells (MoDCs) by culturing in R-10 medium (RPMI GlutaMAX #61870010, 10% FBS, pen-strep) supplemented with 100 ng/mL GM-CSF and 40 ng/mL IL-4, refreshed every 3 d, in non-treated dishes. Non-adherent and semi-adherent cells were harvested and used between days 7 and 10 of culture, and their purity was confirmed to be  > 80% CD11c^+^HLA-DR^+^using flow cytometry. Maturation and activation levels were evaluated by CD80/86 and CD40 expression. Primary naïve CD4^+^ and CD8^+^ T cells, or total T cells, were obtained from healthy donor PBMCs by negative selection using the EasySep Human Naïve CD4 or CD8 T cell isolation kit (#17555 and #17968, StemCell), or EasySep Human T cell isolation kit (#17951, StemCell), and maintained in R-10 medium supplemented with 100 U/mL rhIL-2 (Proleukin).

#### *Antigen-specific CD8* ^*+*^ *T cell clones*

Melan-A/MART-1 specific CD8^+^ T cell clones were purchased (HD358 #ASTC-1072, HemaCare Cellero, Charles River) or generated *in vitro* (5B3) as previously described.^44^ Briefly, Melan-A/MART-1 specific CD8^+^ T cells were purified from healthy donor PBMCs by cell sorting using MHC class I/peptide (Melan-A/MART-1 26-35 ELAGIGILTV) tetramers and cloned by limiting dilution culture in the presence of 1 µg/mL Phaseolus Vulgaris Leucoagglutinin (PHA-L, #L-1110-5, Vector Laboratories), feeder cells (allogeneic irradiated PBMCs), and 150 U/mL hrIL-2. Clones were restimulated periodically (every 2–3 weeks) by co-culture with PHA-L and allogeneic irradiated PBMCs. At the end of the expansion, cells were tested for tetramer positivity (tetramers were kindly provided by Immatics Biotechnologies GmbH) using flow cytometry. The response to Melan-A/MART-1 peptide (#GM-PT001, Proteogenix) was measured by IFNG ELISA according to the manufacturer’s instructions (#88-7316-88, ThermoFisher). Clones were expanded in CTL medium (DMEM 25 mM HEPES, pen-strep, 8% HS, NEAA, sodium pyruvate) and then maintained in R-10 +  medium (RPMI GlutaMAX, 10 mM HEPES, pen-strep, 10% FBS (or 8% HS), NEAA, sodium pyruvate, 100 U/mL rhIL-2).

### Antigens

#### Phagocytic beads

Ovalbumin-coated beads (OVAb) were prepared as follows: 3.0 µm carboxyl polystyrene microspheres (#CP-30-10, Spherotech) were washed three times in PBS, activated with 50 mM 1-Ethyl-3-(3-dimethylaminopropyl)carbodiimide hydrochloride (EDC-HCl) in PBS for 15 min at RT, followed by three washes in borate buffer (0.1 M Na_2_B_4_O_7_). Activated beads were then incubated with 10 mg endotoxin-free chicken ovalbumin (OVA, #vac-pova-100, InvivoGen) in borate buffer overnight at 4°C with shaking. Activation with EDC and covalent coupling with OVA were repeated three times. The following day, the beads were washed twice with 250 mM glycine in PBS and twice with PBS. The beads were either left unopsonized or opsonized with anti-OVA. Finally, beads were resuspended in PBS+1% pen-strep, counted using a Countess II automatic counter, and used at a ratio of 50:1 or 10:1 with the number of DCs, as indicated below.

#### Tumor lysate

B16-OVA cells were trypsinized, washed with PBS, resuspended at a concentration of 1 × 10^7^ cells/mL, and subjected to six consecutive freeze-thaw cycles (3 min in liquid nitrogen and 3 min at 56°C). The number of particles was determined using a Countess II automatic counter (ThermoFisher). Lysates (Tlys) were aliquoted and stored frozen at −80°C and used at a ratio of 1:1 particles to the number of DCs unless otherwise stated.

#### Soluble peptides

The following peptides were used: 1 µg/mL OT-I peptide (OVA 257-264, SIINFEKL) (#vac-sin, Invivogen), 5 µg/mL OT-I long peptide (OT-I-L): (Pam2)-KMFVESIINFEKL (custom-synthesized, Synthetic Biomolecules).^45^ The following long(L) and short (S) peptides were custom-synthesized by ProteoGenix: 10 µM Melan-A/MART-1 16-40 long peptide (MLANA-L) GHGHSYTTAEELAGIGILTVILGVL, 1 µg/mL gp100-L 202–222(T210M) SSSAFTIMDQVPFSVSVSQL, 1 µg/mL NY-ESO-1-L 157–170(C165V) SLLMWITQCFLPVF, 1 µg/mL: Melan-A-S 26–35(A27L) EAAGIGILTV, 1 µg/mL gp100-S 209–217(T210M ) IMDQVPFSV. NY-ESO-1-S 157–165(C165V) SLLMWITQV used at 1 µg/mL was purchased from Iba Lifesciences.

### Store-operated calcium entry (SOCE) modulating compounds

The following SOCE modulating compounds were used: Tg, at 0.05 or 0.1 µM unless otherwise specified, 2-APB at 16 µM, CPA at 1 µM, PAF at 2 µM. Synthetic 2-APB analogs (synthesized in-house^22^) were applied at 5 or 10 µM, unless otherwise specified. 2-APB analogs are named according to their position (*para-ortho-meta*-) and elements of the modification with respect to the 2-APB parental molecule: *p*-Br, *para*-bromo; o-Cl, *ortho*-chloro; *p*-F, *para*-fluoro; o-Br, *ortho*-bromo; Trp, (5-HO-Trp) l-5-Hydroxytryptophan; o-F, *ortho*-fluoro; *p*-SO2, (*p*-SO2NMe2) *para*-dimethylsulfamoyl; m-F, *meta*-fluoro; Met, l-methionine.

### Dendritic cell (DC) maturation, antigen-loading, and SOCE modulator treatment

Murine DCs (JAWSII or BMDCs) were matured (or not) overnight with 1 µg/mL lipopolysaccharide (LPS, #00-4976-93, ThermoFisher). Human MoDCs were matured overnight with 5 ng/mL IL-1ß, 15 ng/mL IL-6, and 20 ng/mL TNFA (#200-01B, #200-06RC, and #300-01A; Peprotech). 16 h later, DCs were loaded with antigens (phagocytic beads, soluble peptides, or tumor cell lysate), followed by the addition of the SOCE-modulating compound. Controls included either no compound or anhydrous DMSO (vehicle) at the highest concentration used for the tested compounds. Cells were incubated for 4 h, after which the supernatant was discarded or collected for cytokine analysis (cytokine array), and DCs were washed and used for downstream analysis or co-cultured with T cells.

### *In vitro* DC-T cell co-cultures

#### Cytokine production and proliferation assays

DCs were matured, loaded with antigens, and treated with SOCE modulators, as described above. 4 h after antigen and compound addition, DCs were washed in DC medium, and T cells were added at a ratio of 2:1 to DCs. The cells were then incubated at 37°C/5% CO2. Supernatants were collected at 24, 48, and 72 h for the evaluation of cytokine production by ELISA according to the manufacturer’s instructions (IL-2 and IFNG ELISA kits, #88-7024-86 (mouse IL-2), #88-7025-88 (human IL-2), #88-7314-88 (mouse IFNG), #88-7316-88 (human IFNG), ThermoFisher). The absorbance was measured using a Ledetec96 plate reader (Labexim Products). T cell proliferation was evaluated by bromodeoxyuridine incorporation using the Cell Proliferation ELISA, BrdU (Chemiluminescent) kit (#11647229001, Roche), according to the manufacturer’s instructions, where BrdU was added to the culture medium 8 h prior to the experiment endpoint. Horseradish peroxidase substrate was added using a Zephir automatic liquid handler workstation (Perkin Elmer), and luminescence was measured using a SpectraMax L plate reader (Molecular Devices). To correct for inter-experimental variability in baseline signal intensity, data were normalized within each experimental day as described in the “Statistics” section.

To determine the cellular source of cytokine production in the co-cultures, intracellular cytokine staining (ICS) was performed after co-culture of treated MoDCs with autologous T cells at a 1:2 ratio. MoDCs were differentiated, matured, loaded with antigens, and treated with SOCE modulators as described above, except that cells were loaded with a peptide pool containing Melan-A, gp100, and NY-ESO-1 peptides, either as long peptides requiring intracellular processing before presentation or as short HLA-A2-compatible minimal peptides. Treatments included DMSO vehicle control, Tg (0.1 µM), pBr (10 µM), or ionomycin (0.1 µM).

T cell proliferation was additionally evaluated by CellTrace Violet (CTV) dilution. Briefly, autologous total T cells were stained with 2.5 µM CTV in PBS for 20 min at 37°C, followed by dilution 1:5 in full medium and an additional 20 min incubation at 37°C. Cells were then washed and added to MoDC cultures after antigen loading, SOCE modulator treatment, and washing of the MoDCs. Cells were incubated at 37°C/5% CO2 for 48 h. Brefeldin A (GolgiPlug, BD Biosciences, #555029) was added at 1 µL/mL 16 h prior to the end of the incubation. ImmunoCult Human CD3/CD28 T Cell Activator (#10971, StemCell) was used as a positive control for cytokine expression. Fluorescence-minus-one (FMO) control was used as a negative cytokine expression. CTV-labeled T cells without MoDC co-culture were used to define non-proliferating or background CTV dilution. CTV-unlabeled T cells without MoDC co-culture were used to define 100% CTV-dilution. At the endpoint, cells were stained for flow cytometry as described in the “Flow Cytometry” section. Samples were acquired on an LSRFortessa flow cytometer (BD Biosciences) and analyzed using FlowJo software.

### Resazurin reduction toxicity assay

25,000 BMDCs per well were seeded in round-bottom non-tissue culture-treated 96-well plates. Cells were matured, loaded with antigens, and treated with SOCE modulators, as described above. After 4 h, the cells were washed with complete medium. 20 h later, 15 µg/mL resazurin was added to each well, and the cells were further incubated for 4 h. At the end of the incubation period, the plates were centrifuged, and the supernatant was transferred to a black clear-bottom 384-well plate to measure fluorescence (Ex560nm-Em600nm) using a SpectraMax Paradigm plate reader (Molecular Devices). To correct for inter-experimental variability in baseline signal intensity, data were normalized within each experimental day as described in the “Statistics” section. Relative viability was calculated by dividing the normalized fluorescence value of each well by the average normalized fluorescence value of untreated control cells from the same experiment and multiplying by 100.

### Calcium signaling measurement

BMDCs were loaded with 4 µM Fluo-8-AM (AAT Bioquest) for 15 min at RT, followed by 15 min at 37ºC and 15 min at RT, in modified Ringer’s solution (140 mM NaCl, 5 mM KCl, 1 mM MgCl2, 20 mM HEPES, 1 mM CaCl2, 10 mM glucose) containing 500 µM sulfinpyrazone and 0.04% pluronic. At the end of the incubation period, the cells were resuspended in modified Ringer’s solution with 500 µM sulfinpyrazone, and 10,000 cells per well were seeded in a black optical clear-bottom 384-well plate. Total calcium signals were measured by adding SOCE modulators directly to the well after 2 min of baseline fluorescence (490/525 nm) acquisition in calcium-containing Ringer’s solution. The ER calcium release and intracellular calcium influx components of the total calcium signal were measured using a two-step protocol. First, cells were exposed to 2 mM EGTA to chelate extracellular calcium (calcium-free medium). After 5 min of incubation, SOCE modulators were added, and fluorescence was measured for 8 min to evaluate the emptying of ER calcium stores. Subsequently, the calcium influx was measured by adding extracellular calcium (4 mM CaCl2). To evaluate the contribution of ORAI or IP3R channels to the signals, the ORAI inhibitor GSK-7975A^23^ and the IP3R antagonist xestospongin C^24^ were added 5 min prior to the addition of the SOCE modulators. All measurements were performed using an FDSS microCELL plate reader (Hamamatsu) equipped with a built-in robotic pipettor for the addition of EGTA, the compound, and CaCl2 solutions. The fluorescence values were analyzed using MS Excel. Normalized fluorescence values are expressed as the F/F0 ratio, where F0 is defined as the average well fluorescence value for 1 min prior to solution change. The area under the curve (AUC) and maximum (peak) F/F0 values were calculated using Prism v10.0 (GraphPad).

### Immunofluorescence

BMDCs were seeded on 12-mm round #1.5 coverslips in 24-well plates at a density of 2 × 10^5^ cells/well, matured, loaded with antigens, and treated with SOCE modulators, as described above. After 4 h, the plate was placed on ice for 5 min, and the supernatants were removed and replaced with a 1:1 volume of 4% PFA/PBS solution for anti-OVA staining or with 1%PFA/PHEM solution (PHEM: 60 mM PIPES, 25 mM HEPES, 10 mM EGTA, 2 mM MgCl_2_, pH 6.9) for anti-MHC-I-*p* staining for 10 min at RT. The cells were then washed with 0.1% NaBH4/PBS solution for 1 min, washed 2x in PBS, permeabilized in 0.1% Triton-X100/PBS for 5 min, and washed with 3x PBS for anti-OVA staining. For anti-MHC-I-*p* staining, permeabilization was achieved by adding 0.05% saponin at every step, except for PBS washes. Coverslips were transferred to a humid chamber and blocked in ImageIT-FX (#I36933, ThermoFisher) for 30 min, in 2% BSA/PBS for 30 min, and incubated overnight at 4 °C with rabbit-anti-OVA diluted in 0.2% BSA/PBS or mouse anti-MHC-I-*p*. The coverslips were washed 3x in PBS and incubated in AF488-GAR or AF488-GAM for 1 h at room temperature. The cells were washed 3x in PBS and incubated with 1 µg/mL Hoechst 33342/PBS for 5 min prior to the final wash. Coverslips were mounted in Prolong Diamond anti-fade (#P36970, ThermoFisher) and sealed with nail polish. Imaging of brightfield, Hoechst (Ex405:Em460/50 nm), and AF488 (Ex488:Em525/50 nm) was performed using a Nipkow Yokogawa Nikon spinning disk confocal imaging system equipped with a Plan Apo 63x/1.4 Oil DICIII objective and Visiview software (Visitron Systems). Image analysis and mean cellular OVA or MHC-I-*p* (AF488) fluorescence intensity (MFI) were quantified by manually defining the total cell regions of interest at a single mid-nuclear plane using the brightfield and Hoechst channels with ImageJ 1.52p software (National Institutes of Health, NIH, USA).

### Antigen transfer

BMDCs were collected, counted, and labeled or not with 1 µM CTV in PBS for 20 min at 37°C/5%CO_2_. Cells were diluted 1:5 in full medium, incubated for another 20 min at 37°C/5%CO_2,_ washed, and resuspended in full medium. CTV-labeled and unlabeled cells were then seeded in 96-well round-bottom plates at 1 × 10^5^ cells/well, and matured with LPS overnight. Matured donor BMDCs were loaded with 100 µg/mL OVA-AF647 and treated or not with DMSO vehicle, Tg (0.1 µM), or pBr (10 µM) for 4 h. After incubation, donor cells were washed and co-cultured 1:1 with antigen-naïve recipient BMDCs overnight to allow antigen transfer from OVA-loaded donors to unloaded recipients. Donor and recipient populations were distinguished by CTV labeling, with reciprocal CTV-labeled and unlabeled combinations included where indicated. CTV-labeled and unlabeled cells exposed or not to OVA were also kept in separate cultures as controls. After 24 h co-culture, cells were stained for flow cytometry as described in the “Flow Cytometry” section using an LSRFortessa flow cytometer (BD Biosciences) and analyzed using FlowJo software. Antigen transfer was quantified based on AF647 signal detection within the recipient cell population.

### *In vitro* migration

1.2 to 2 × 10^5^ LPS-matured BMDCs were treated with SOCE modulators for 4 h, before washing and resuspension in 400 µL phenol-free serum-free medium (migration medium, DMEM high-glucose HEPES #21063029) and transferred to the upper layer of an 8 µm pore polycarbonate membrane insert (Corning). Transwell inserts were placed in a 24-well plate containing 400 µL migration medium supplemented (or not) with 100 ng/mL CCL21 (#250-13, Peprotech). The cells were incubated overnight at 37 °C/5% CO2. The following day, the migrated cells were recovered from the bottom wells, labeled with 2.5 µM DRAQ5, and counted by flow cytometry. Values were normalized by experimental day as described in the “Statistics” section. Relative migration, expressed as the transwell migration index, was calculated by dividing the migrated cells in each condition by the average of migrated cells in the untreated (CTR) condition.

### *In vivo* migration

BMDCs from CD45.1 mice were matured with LPS, loaded or not with 5 µg/mL OT-I-L peptide, and treated with SOCE modulators, DMSO vehicle control, or left untreated, as described above. SOCE or DMSO-treated cells (antigen-loaded or not) were stained with 5 µM CFSE for 10 min at 37°C and washed twice in BMDC medium, whereas matched untreated control cells were left unstained. CFSE-stained treated cells and their corresponding untreated unlabeled controls were mixed at a 1:1 ratio, and 1 × 10^6^ total cells in 20 µL PBS were injected subcutaneously into the footpads of anesthetized CD45.2 recipient mice. 24 h later, the mice were euthanized, and the popliteal lymph nodes (pLN) were harvested. Cell migration was evaluated by flow cytometry gating on CD45.1 and CD11c markers, and CFSE was used to distinguish SOCE modulator- or DMSO-treated cells from co-injected untreated control cells.

### *In vivo* DC-mediated T cell activation

Naïve CD8^+^ OT-I T cells were labeled with 5 µM CFSE for 10 min at RT and washed three times. Cells were adjusted to a concentration of 2 × 10^7^ cells/mL in PBS, and 100 µL of CFSE-labeled cells were injected into the tail vein of CD45.2 recipient mice. CD45.1 BMDCs were matured with LPS, loaded or not loaded with the OT-I-L peptide, and treated with SOCE modulators or DMSO as described above. 24 h after OT-I T cells were injected, the mice were anesthetized and subcutaneously injected into the footpad with 1 × 10^6^ BMDCs in 20 µL PBS. 48 h later, “draining” (dLN) and contralateral (CtrlLN) popliteal lymph nodes were harvested, and CFSE dilution, total number of CD45.1^+^ CD8^+^ OT-I cells, as well as their activation (measured as CD25 and CD69 surface marker expression), were analyzed by flow cytometry. To correct for inter-experimental variability in baseline signal intensity, data were normalized within each experimental day as described in the “Statistics” section.

### *In vivo* tumor progression and DC vaccine

WT recipient mice were anesthetized and inoculated with 2.5 × 10^5^ B16-OVA tumor cells in 100  µL PBS by intradermal injection into their right flank. The tumor surface was monitored every 2–3 d using a caliper and calculated using the formula length × width. When tumors reached 20–25 mm^2^, mice were allocated using stratified randomization: tumors were ranked by size, and animals were assigned in sequence to evenly distribute sizes across groups. The operator administering DC vaccines was aware of group allocation; tumor measurements were performed blind to treatment. Flow-cytometry analyses at endpoint were conducted after unblinding at sacrifice. The mice were anesthetized and received the first dose of DC vaccine via subcutaneous injection into the footpad. DC vaccines consisting of 1 × 10^6^ BMDCs matured with LPS, loaded with antigens, and treated with SOCE modulators or DMSO control, as described above, were resuspended in 20 µL PBS. Mice were injected with PBS as unvaccinated controls. Six to seven days later, the mice were anesthetized and received a second dose of the same DC vaccine or PBS injection. All tumor-bearing mice were monitored daily and euthanized when tumors reached a maximum diameter of 15 mm or an area > 250 mm^2^, or if ulceration, necrosis, or distress was observed, in accordance with approved humane endpoints. Attrition was therefore limited to animals reaching humane endpoints or dying unexpectedly before study completion. These cases were included in tumor-growth analyses but were unavailable for endpoint assays (e.g., flow cytometry). Potential confounders such as cage location and measurement order were not controlled; measurement order was rotated where feasible. For immune-profiling, surviving mice were analyzed 28 d after tumor inoculation. For survival analyses, mice received a third DC vaccine or PBS 6–7 d after the second dose and were monitored until humane endpoints.

### Tissue processing

For *in vivo* migration and *in vivo* T cell responses experiments, popliteal lymph nodes (pLN) were crushed and filtered through a 70 µm mesh filter, washed twice in FACS buffer, and subsequently stained with antibodies. For the DC vaccination studies, tumors, pLN, and tumor-draining inguinal lymph nodes (iLN) were cut into small pieces with scissors, digested in 1 mg/mL collagenase D (#COLLAD-RO, Roche) and 10 µg/mL DNase I (#10104159001, Roche) in HBSS (#14175053, ThermoFisher) for 40 min at 37°C, and then mixed up and down every 10 min. The digested cell solutions were filtered through a 70 µm strainer and washed once in FACS buffer. Tumor samples were transferred to a new tube containing Lympholyte cell separation medium (#CL5035, Cedarlane), centrifuged, and the interphase containing living cells was collected and washed once in FACS buffer. All cells were counted, and 2 × 10^6^ cells per sample were analyzed using flow cytometry.

### Flow cytometry

Single-cell suspensions were incubated with Fc-Block for 15 min at 4°C and then stained with antibodies in PBS or FACS buffer for 20–30 min at 4°C, protected from light during incubations. For intracellular cytokine staining, when required, cells were first restimulated in complete RPMI containing 100 ng/mL PMA, 1 µg/mL ionomycin, and GolgiStop solution (1/1000, #51-2301KZ, BD Biosciences) for 4 h at 37°C/5% CO_2_. Intracellular staining was then performed using the intracellular Fixation and Permeabilization Buffer Set (Fix/Perm Concentrate (#00-5123-43, ThermoFisher), Fix/Perm Diluent (#00-5223-56, ThermoFisher), permeabilization buffer (#00-8333-56, ThermoFisher), and stained for 45 min at RT protected from light. To extrapolate the total cell numbers, counting beads (#C36995, ThermoFisher) were added immediately before running the samples. Data were acquired using a Fortessa analyzer (BD) or a Cytoflex S (Beckman) and analyzed using FlowJo v10.10.0 software.

### Bulk RNAseq

1 × 10^6^ MoDCs from three different healthy donors were matured overnight, loaded with Melan-A antigen, and treated with SOCE modulators or not as a control, for 6 h at 37°C/5% CO2. After incubation, the cells were collected, washed once, and resuspended in the RNA lysis buffer. Total RNA was extracted using the Monarch Total RNA miniprep kit (#T2010G, New England Biolabs) according to the manufacturer’s instructions. RNA quantification was performed using a Qubit fluorimeter (ThermoFisher), and RNA integrity was assessed using a Bioanalyzer (Agilent Technologies). The Illumina Stranded mRNA Prep kit (Illumina) was used for library preparation, with 200 ng of total RNA as input. Library molarity and quality were assessed using the Qubit and Tapestation analyzers (Agilent Technologies). Libraries were sequenced on a NovaSeq 6000 sequencer (Illumina) using a 100-bp single-end reads protocol at the iGE3 Genomics Platform of the University of Geneva (https://ige3.genomics.unige.ch/). Quality control was performed using FastQC v.0.11.9. The reads were aligned using STAR v.2.7.10b^46^ to the human Ensembl genome GRCh38. Gene expression was quantified using HTSeq v.0.11.13.^47^ Lowly expressed genes were filtered out, and normalization and differential expression analysis were performed using the R/Bioconductor package edgeR v.3.42.4.^48^ Statistical significance was assessed using a general linear model, negative binomial distribution, and quasi-likelihood F-test. Genes with a fold change > 2 and *p*-value < 0.05 (with multiple testing Benjamini and Hochberg False Discovery Rate correction) were considered differentially expressed. Clustering of differentially expressed genes was performed using hierarchical clustering with Pearson’s correlation distance and average linkage. Over-representation analysis of Gene Ontology terms and KEGG pathways was performed using the R/Bioconductor packages clusterProfiler v.4.9.0 and org.Hs.eg.db 3.15.0. Subsequent pathway analysis was conducted using the iDEP 2.01 online platform (https://bioinformatics.sdstate.edu/idep/accessed 13^th^ June 2024).^49^ Unsupervised pathway enrichment analysis was performed using the Reactome, WikiPathways, and Hallmark MSigDB databases integrated within iDEP, with default parameters except FDR *p*-value cutoff set at 0.05 and maximum pathways set at 950. Word frequency analysis was performed using Word Frequency Analyzer (https://wordfrequency.org/accessed June 10, 2025), Text analyzer (https://www.online-utility.org/text/analyzer.jsp accessed June 10, 2025), and Word Cloud Generator ETHZ (https://wordclouds.ethz.ch/accessed June 10, 2025). To facilitate biological interpretation and reduce dimensionality, pathway names were further analyzed using keyword frequencies and visualized using word clouds. To support the interpretation, enriched pathways were categorized manually based on recurring keywords and biological themes. These categories are listed in Data S1 (Pathway Summary tab) along with the associated keyword criteria. Color coding was consistently applied across all pathway analysis sheets to indicate category membership. The lists of selected secreted factors generally associated with T-cell activation and inhibition were drafted from,^29^ ^,^ ^33^ where factors associated with both activation and inhibition were excluded from the lists. The lists of selected co-stimulators and co-inhibitory molecules were drafted from previous studies.^3^ ^,^ ^30^ Targeted gene set enrichment analysis (GSEA) was performed using GSEA v4.3.3^50^ with default parameters, with the exception of the maximum gene set size set to 705, and the following selections: collapse to gene symbols, gene_set permutation type, and chip platform Human_Ensembl_Gene_ID_MSigDB.v2024.1. Hs.chip. The custom gene set input file for the analysis, output gene ranks, and output GSEA reports are provided in Data S1. Input gene sets included: genes associated with the human DC1, DC2, and mregDC phenotypes described in^32^; genes associated with the type I IFN - ISG stimulatory DC vaccine phenotype T1, a phenotype showing positive correlation with the clinical effect of DC vaccination; suppressive mregDC vaccine phenotype T3, a phenotype showing negative correlation with the clinical effect of DC vaccination^31^; genes upregulated or downregulated upon stimulation of immature DCs with LPS and IFNG; genes of factors secreted upon stimulation of immature DCs with LPS and IFNG; and genes of factors secreted upon stimulation of immature DCs with CD40L.^33^ Gene sets showing FDR q-value < 0.25 were considered significantly enriched.

### Cytokine array

BMDCs were seeded and treated as described in the immunofluorescence (IF) section. After 4 h of treatment, the plates were placed on ice for 5 min, and the supernatant was collected. A Proteome Profiler Mouse Cytokine Array kit, Panel A (#ARY006, R&D Systems), was then performed according to the manufacturer’s instructions using 200 µL of BMDC supernatants collected from BMDCs derived from 3 to 4 different mice that were pooled and diluted to 1 mL using the kit’s supernatant dilution solution before the addition of the capture antibody mix. For experiments with human MoDCs, 1 × 10^5^ cells were seeded in duplicate wells in 96-well round-bottom plates and matured overnight, and treated or not with DMSO or Tg as above. Supernatants of duplicates and of 3 different donors were collected after 24 h and pooled for analysis with the Proteome Profiler Human Cytokine Array kit. The array membranes were incubated overnight, and detection was performed using Immobilon Western Chemiluminescent HRP Substrate (Merck, #WBKLS0050). Images were captured every 10 s for 2 min using an LAS-2000 imager (Fujifilm). Densitometry analysis was performed on the images after 1 min for spots using manually defined regions of interest using ImageJ v. 1.52p software (NIH). Relative luminescence units (RLU) were calculated for each spot by first subtracting the background (mean of PBS/background spots) and then dividing the chemiluminescence intensity signal of each cytokine spot by the mean of all cytokine spots across all arrays analyzed in the same image (four arrays per image).

### Statistical analysis

All experiments were performed with at least two independent biological replicates, unless otherwise specified. Technical replicate wells (typically 2–3 per condition, depending on the assay) were averaged before analysis and were not treated as independent observations. To correct for inter-experimental and/or inter-donor variability in baseline signal intensities, data were normalized by experimental day and/or biological donor, where indicated. For each independent experiment and/or donor, the mean signal across all conditions was first calculated. A global mean across all experimental days was then determined. A normalization factor for each experiment and/or donor was calculated by dividing the global mean by the mean of the corresponding experimental day and/or donor. Raw values from each condition within a given experiment were subsequently multiplied by the corresponding normalization factor. This normalization corrected for day-to-day and/or donor-to-donor variations in overall signal intensity while preserving relative differences between experimental conditions within each independent experiment. The number and nature of biological replicates and technical replicates are indicated in the figure legends. Initial compound screening experiments performed once with technical replicates are indicated as exploratory. The experimental unit was the individual animal for *in vivo* studies and for mouse primary or *in vitro-*derived mouse cell assays. For human cell assays, the experimental unit was an independent donor. Primary outcomes were tumor area over time for tumor-growth studies, time to humane endpoint for survival studies, and prespecified flow cytometry readouts for mechanistic assays, including CFSE dilution, activation markers, cell migration to dLNs, and predefined immune-cell frequencies/activation indices by flow cytometry in tumor and lymph nodes. Sample sizes were estimated based on prior experience, feasibility, expected variability, and the 3R principles. *In vitro* assays were typically performed with 3–6 independent donors or animals, with technical triplicates where possible. *In vivo* group sizes were guided by previous studies using the B16 melanoma model and adjusted across cohorts to account for expected variability and animal losses. Tumor-growth and immune-profiling experiments included up to 25 animals per group, across 1-3 independent cohorts. Survival experiments included 10 animals per group, and shorter-term mechanistic assays, such as migration assays, used 3 animals per group. The study was not powered to detect sex differences. Statistical analyses were performed in GraphPad Prism v10 (GraphPad Software Inc., USA) with *n* defined as the number of independent biological samples. For datasets with sufficient biological replicates, normality was assessed using the Shapiro–Wilk test and equality of variances using Brown–Forsythe and Bartlett’s tests, as appropriate. For small-*n* experiments, typically *n* = 3–5, formal normality testing was not considered informative; parametric tests were therefore used when appropriate for the experimental design, assuming approximately normally distributed biological replicate means. Two-group comparisons were analyzed using paired or unpaired two-tailed t-tests, as appropriate. Multiple-group comparisons were analyzed using one- or two-way ANOVA followed by Tukey’s test for all-group comparisons or Dunnett’s test for comparisons to a shared control. When assumptions were not met, Kruskal–Wallis tests with Dunn’s multiple-comparison test, Welch’s correction, Brown–Forsythe and Welch ANOVA, or mixed-effects models were used as appropriate. The statistical comparisons shown correspond to planned comparisons relevant to the experimental question.

## Supplementary Material

Supplementary_ARRIVE_Checklist Supplementary_ARRIVE_Checklist.pdf

CruzCobo_2026_Supplementary_Figure_Legends.docx CruzCobo_2026_Supplementary_Figure_Legends.docx

FigureS1.jpg FigureS1.jpg

FigureS2.jpg FigureS2.jpg

FigureS3.jpg FigureS3.jpg

FigureS4.jpg FigureS4.jpg

FigureS5.jpg FigureS5.jpg

FigureS6.jpg FigureS6.jpg

FigureS7.jpg FigureS7.jpg

FigureS8.jpg FigureS8.jpg

FigureS9.jpg FigureS9.jpg

FigureS10.jpg FigureS10.jpg

FigureS11.jpg FigureS11.jpg

FigureS12.jpg FigureS12.jpg

## Data Availability

Data associated with this study are available in Yareta, the University of Geneva’s FAIR-compliant data repository, under a CC BY-NC-ND 4.0 license: https://doi.org/10.26037/yareta:xi4mah62rranhp63ylplop33qy. The RNA-sequencing data have been deposited in the NCBI Gene Expression Omnibus (GEO) under accession number GSE342540. Any additional data supporting the findings of this study are available from the corresponding author upon reasonable request.
