# Supplementary material for: Store-operated calcium signaling modulators improve a dendritic cell-based vaccine against melanoma
Source: Oncoimmunology. 2026 Aug 28;15(1):2723541. doi: 10.1080/2162402X.2026.2723541 (PMC13531994; doi:10.1080/2162402X.2026.2723541)
Supplement: Supplementary file 2 [file KONI_A_2723541_SM3862.docx]

**Cruz Cobo et al. 2026**

**Supplementary Figure Legends**

**Fig. S1. Tg enhances DC-mediated T cell responses across multiple murine assay systems. A)** IL-2 levels measured by ELISA in supernatants after 24 h co-culture of B3Z or OT-I CD8⁺ T cells with LPS-matured DCs loaded with OVAb, OT-I-L, or Tlys and treated with Tg (purple) or DMSO vehicle control (grey). The DC:T cell combinations are indicated in the graph headers (n = 2 independent biological samples). **B)** IL-2 levels measured by ELISA in supernatants 24 h after co-culture of OT-I CD8⁺ T cells with immature, OVAb-pulsed, Tg-treated BMDCs (n = 4 independent biological samples). **C)** IL-2 levels measured by ELISA in supernatants 24 h after co-culture of OT-II CD4⁺ T cells with mature or immature, OVA-loaded, Tg-treated BMDCs (n = 4 independent biological samples). **D)** Chemical structure and CAS number of *para*-bromo-2-APB (pBr). Bars are means + SEM. Each dot in the bar graphs represents one biological replicate, corresponding to the average of 2–3 technical replicate wells. Data in A-C were normalized within each experimental day to the mean response across all conditions measured in that experiment. Significant p-values were calculated using two-way ANOVA with Sidak’s post-hoc test in A and C, or paired t-test in B, and are shown above bars when significant.

**Fig. S2. Flow cytometry gating strategy for *in vivo* BMDCs migration and DC-mediated T cell activation assays. A)** Gating strategy for analysis of CD45.1⁺CD11c⁺CFSE⁺ BMDCs migrating to popliteal lymph nodes, corresponding to Fig. 2C. **B)** Gating strategy for analysis of OT-I CD8⁺ T cell proliferation, measured by cell count and CFSE dilution, and activation, measured by CD25⁺CD69⁺ expression, corresponding to Fig. 2D-E and Fig. S2C. OT-I cells were identified as CD8⁺CD45.1⁺ cells. **C)** Surface CD25⁺CD69⁺ expression on OT-I cells recovered from dLN; see also Fig. 2D-E. Mice per group in C, DMSO/Tg/pBr: n = 11/13/12 for DCVAC OT-I-L and n = 7/5/6 for DCVAC CTR from 2 independent cohorts. Bars are means + SEM. Each dot in the bar graphs represents one biological replicate, corresponding to one mouse. Significant p-values were calculated using two-way ANOVA with Dunnett’s post-hoc test and are shown above bars when significant.

**Fig. S3. Impact of SOCE modulators on the efficacy of anti-melanoma DC vaccines loaded with tumor lysate.** WT C57BL/6J mice were inoculated with 0.5 × 10⁶ B16-OVA tumor cells and vaccinated according to the strategy described in Fig. 3. **A)** Tumor growth curve, shown as surface area in mm², for mice vaccinated with DCVAC not loaded with antigen and treated with SOCE modulators Tg or pBr, DMSO-vehicle control, or left unvaccinated (PBS). Arrows indicate vaccination. **B-O)** Mice were vaccinated with DCVAC loaded with tumor lysate (Tlys) and treated with SOCE modulators, DMSO-vehicle control, or left unvaccinated (see also Fig. 3). **B)** Proliferation (Ki67⁺) of tumor or tumor-associated cells (CD45⁻). **C)** Frequencies of T cell (TCR⁺) subsets in draining popliteal (pLN) and inguinal (iLN) lymph nodes: CD8⁺ T cells, conventional CD4⁺ T helper cells (CD4^+^ Th, FOXP3⁻), and regulatory T cells (Treg, CD4⁺FOXP3⁺). **D)** Ratio of effector T cells to Tregs in tumors, shown as total CD4^+^ Th cells or total CD8⁺ T cells divided by the total number of Treg cells. **E)** Ratio of effector T cells to Tregs in lymph nodes. **F)** Frequency of functional marker expression in CD8⁺ T cells in lymph nodes: proliferation (Ki67⁺), granzyme B (GrzB^+^), IFNG^+^, and terminal exhaustion markers (LAG3⁺PD1⁺TIM3⁺). **G)** Frequency of functional markers expressed in CD8⁺ T cells in tumors: GrzB⁺IFNG⁺ double-positive cells (left) and TNFA⁺ cells (right). **H)** Frequency of functional and subset markers on CD4 Th cells in lymph nodes: proliferation (Ki67⁺), Th1 (IFNG⁺), cytotoxicity (GrzB⁺), and activation (TNFA⁺). **I-J)** Frequency of functional and subset markers on CD4^+^ Th cells in tumors: proliferation (Ki67⁺), activation (TNFA⁺), cytotoxicity (GrzB⁺), Th1 (IFNG⁺), Th17 (IL-17A⁺), Th2 (IL-4⁺), and terminal exhaustion markers (LAG3⁺PD1⁺TIM3⁺). **K)** Frequencies of DCs (CD11c⁺MHC-II⁺) and proliferating DCs (Ki67⁺) in lymph nodes. **L)** Frequencies of tumor DC subsets among CD11c⁺MHC-II⁺ cells: cDC1s (XCR1⁺), cDC2s (CD11b⁺), and monocyte-derived DCs (MoDCs, Ly6C⁺). **M-N)** Marker-intensity analysis of PDL1, TIM3, and CD80 in total tumor DCs (M) and cDC1/cDC2 subsets (N), shown as MFI. **O)** Frequency of activated macrophages (CD80⁺) among tumor macrophages (F4/80⁺CD19⁻). Bars are means ± SEM. Each dot in the bar graphs represents one biological replicate. Mice per group were PBS/DMSO/Tg/pBr n = 5/7/7/7 in A, from one cohort; n = 14/12/15/13 in B, D, G IFNG^+^GrzB^+^, I Ki67⁺, J GrzB⁺ and IFNG⁺ and L, from 2 independent cohorts; n = 6/5/7/6 in C, E, F, H, K, G TNFA^+^, and I TNFA⁺; and n = 8/7/8/7 in J IL-17A⁺, LAG3⁺PD1⁺TIM3⁺, and IL-4⁺, M-O, from one cohort. Significant p-values were calculated using one-way ANOVA for tumor analyses or two-way ANOVA for lymph node analyses, with Tukey’s post-hoc tests, or Kruskal-Wallis tests with Dunn’s post-hoc tests when normality assumptions were not met. Exact p-values are indicated when significant.

**Fig. S4. Impact of SOCE modulators on the efficacy of anti-melanoma DC vaccines loaded with OT-I-L peptide.** WT C57BL/6J mice were inoculated with 0.5 × 10⁶ B16-OVA tumor cells and vaccinated according to the strategy described in Fig. 3. Mice were vaccinated with DCVAC loaded with OT-I-L peptide and treated with SOCE modulators Tg (purple) or pBr (yellow), DMSO-vehicle control (cyan), or left unvaccinated (PBS, grey). **A)** Tumor growth curve, shown as surface area in mm². Arrows indicate vaccination. **B)** Tumor weight at endpoint, in grams. **C)** Frequency of leukocyte infiltration, shown as CD45⁺ cells in tumors. **D)** Frequencies of T cell (CD3^+^) subsets in tumors: CD8⁺ T cells, conventional CD4⁺ T helper cells (Th, CD4^+^FOXP3⁻), and regulatory T cells (Treg, CD4⁺FOXP3⁺). **E)** Ratio of effector T cells to Tregs in tumors, shown as total CD8⁺ or CD4^+^ Th cells divided by the total number of Treg cells. **F)** Frequency of functional markers on CD8⁺ T cells in tumors: proliferation (Ki67⁺), activation (IFNG⁺, TNFA⁺), cytotoxicity (GrzB⁺); and density of GrzB-producing cells, shown as number of cells per gram of tumor. **G)** Frequency of functional and subset markers on Th cells in tumors: proliferation (Ki67⁺), Th1 (IFNG^+^), cytotoxicity (GrzB⁺), and activation (TNFA⁺). **H)** Frequency of myeloid-derived suppressor cells in tumors: monocytic MDSCs (F4/80⁻CD19⁻CD11b⁺Ly6C⁺) and polymorphonuclear MDSCs (PMN-MDSCs, F4/80⁻CD19⁻CD11b⁺Ly6G⁺). **I)** Frequencies of total DCs (CD11c⁺MHC-II⁺), DC subsets among total DCs, cDC1s (XCR1⁺), cDC2s (CD11b⁺), and monocyte-derived DCs (MoDCs, Ly6C⁺), and proliferating DCs (Ki67⁺) in tumors. **J)** Frequencies of T cell (CD3^+^) subsets in footpad- and tumor-draining lymph nodes, pLN and iLN, respectively. **K)** Frequency of functional markers on CD8⁺ T cells in lymph nodes. **L)** Frequency of functional and subset markers on CD4^+^ Th cells in lymph nodes. **M)** Frequency of MDSCs in lymph nodes: monocytic MDSCs and PMN-MDSCs. **N)** Frequencies of DCs, their subsets cDC1s and cDC2s, and proliferating DCs in lymph nodes. Bars are means ± SEM. Each dot in the bar graphs represents a biological replicate. Mice per group, all from a single cohort, are in A-B: PBS/DMSO/Tg/pBr n = 7/6/7/7. Mice per group in C-I: PBS/DMSO/Tg/pBr n = 7/6/7/6. Mice per group in J-N: PBS/DMSO/Tg/pBr, n = 6/5/7/7 for pLN and n = 7/5/6/7 for iLN. Significant p-values were calculated using one-way ANOVA for tumor analyses or two-way ANOVA for lymph node analyses, with Tukey’s post-hoc tests, or Kruskal-Wallis tests with Dunn’s post-hoc tests when normality assumptions were not met. Exact p-values are indicated beside (A) or above (B-N) bars when significant.

**Fig S5: Flow cytometry gating strategy for the identification and phenotypic characterization of immune populations shown in Fig. 3, S3 and S4.** Representative plots corresponding to a tumor sample from a mouse vaccinated with SOCE-treated DCVAC are shown. **A) Lymphoid panel:** Cells were gated based on forward and side scatter (FSC-A × SSC-A), together with beads used for quantification of cell numbers, followed by singlet discrimination (FSC-H × FSC-A) and exclusion of dead cells using viability dye (CD45 × LIVE/DEAD Aqua). From live singlets, leukocytes (CD45⁺) and tumor or tumor-associated cells (CD45⁻) were gated. Within CD45⁻ cells, proliferation (Ki67⁺) was measured. Within leukocytes, TCR or CD3 markers were used to distinguish T cells, and from the TCR⁻ or CD3⁻ gate, NK cells were identified as NKP46⁺ and further analyzed for intracellular IFNG and GrzB expression. TCR⁺ or CD3⁺ cells were subdivided into CD4⁺ and CD8⁺ T cells. The CD8⁺ T cell subset was analyzed for functional and phenotypic markers, including proliferation (Ki67⁺), activation (IFNG^+^), cytotoxicity (GrzB), and terminal exhaustion markers (LAG3, PD-1, TIM3). CD4⁺ T cells were further divided based on FOXP3 expression into conventional CD4⁺ T cells (Th; FOXP3⁻) and regulatory T cells (Tregs; FOXP3⁺). The Th CD4⁺ T cell subset was analyzed for functional and phenotypic markers, including proliferation (Ki67⁺), Th subset-associated cytokine production or cytotoxicity markers (IFNG^+^ for Th1; GrzB^+^ for cytotoxicity; IL-4^+^ for Th2; IL-17A^+^ for Th17; TNFA^+^ for activation/inflammatory cytokine production), and terminal exhaustion markers (LAG3^+^, PD-1^+^, TIM3^+^). **B) Myeloid panel:** Gating of cells, singlets, and live cells is as in A. Next, CD45⁺ cells were gated for CD19 expression to identify B cells (CD19⁺), which were further evaluated for activation (CD80⁺), MHC-II expression, and co-inhibitory markers (TIM3^+^, PDL1^+^). CD19⁻ cells were gated for F4/80 and CD11b to identify macrophages (MΦ; F4/80⁺CD11b⁺), which were further categorized as M1- or M2-associated subsets based on iNOS⁺ or CD206⁺ expression, respectively, and analyzed for activation (CD80⁺), MHC-II expression, and co-inhibitory markers (TIM3^+^, PDL1^+^). F4/80⁻ cells were gated on Ly6C and Ly6G to identify myeloid-derived suppressor cells, with monocytic MDSCs (Ly6C⁺CD11b⁺) and polymorphonuclear MDSCs (PMN-MDSCs; Ly6G⁺CD11b⁺) further examined. Monocyte-derived dendritic cells (MoDCs) were defined within the Ly6C⁺ population as CD11c⁺MHC-II⁺. Conventional DCs were gated within F4/80⁻ cells as CD11c⁺MHC-II⁺ and further distinguished into cDC1 (XCR1⁺) and cDC2 (CD11b⁺) subsets, and analyzed for activation (CD80⁺) and co-inhibitory markers (TIM3^+^, PDL1^+^). Red arrows indicate the gating hierarchy. Main populations of interest are labeled in red. Red dotted lines indicate downstream phenotypic analyses performed in parallel within the parent population.

**Fig. S6. SOCE modulators induce comparable global calcium signals with distinct influx components in BMDCs.** Fluo-8-loaded BMDCs were treated with SOCE modulators Tg and pBr at the indicated concentrations (in µM), DMSO-vehicle control, or left untreated (CTR), and fluorescence was continuously recorded. Black arrow indicates compound addition. Fluorescence values (F) were normalized to the mean baseline fluorescence 1 min prior to compound addition (F0), the normalized ratio (F/F0) is shown, and the corresponding change in peak fluorescence (ΔPeak) or area under the curve (AUC) are represented in the bar graphs below. **A)** Global calcium signaling in calcium-containing medium after compound addition (n = 5 independent biological samples). **B-C)** As in A), but cells were pre-incubated with B) the Orai inhibitor GSK-7975A (GSK) (n = 5 independent biological samples) or C) the IP3R inhibitor Xestospongin C (Xesto), (n = 3 independent biological samples) for 5 min prior to compound addition. **D)** Intracellular calcium store release measured in calcium-free medium (CF, 5 mM EGTA, n = 3 independent biological samples). **E)** Calcium influx measured upon re-addition of extracellular calcium after treatment in D) (n = 3 independent biological samples). Bars are means + SEM. Each dot in the bar graphs represents one biological replicate, corresponding to the average of duplicate wells. Significant p-values were calculated using one-way ANOVA with Dunnett’s post-hoc test, with comparisons to CTR in A-C or to DMSO in D-E. Exact p-values are shown above bars when significant.

**Fig. S7. SOCE modulators alter antigen retention but do not affect antigen transfer in BMDCs. A)** LPS-matured BMDCs loaded with uncoated beads (CTRb) or OVAb (n = 3 independent biological samples); and OT-I-L, OT-I, or no antigen (CTR), (n = 4 independent biological samples), and treated for 4h with SOCE modulators Tg (0.1 µM, purple) and pBr (10 µM, yellow), DMSO vehicle control (cyan), or untreated (CTR, grey). Mean Fluorescence Intensity (MFI) of surface MHC-I-peptide complexes (MHC-I-p; detected using anti-H-2Kb–SIINFEKL antibody) assessed by FC. **B-C)** Immunofluorescence quantification of intracellular MHC-I-p in B) OVAb- and C) OT-I-L-loaded BMDCs treated or not with DMSO, Tg and pBr as in A) (n = 3 independent biological samples). **D)** Immunofluorescence analysis of residual levels of intracellular OVA antigen (detected using anti-OVA antibody) of cells treated as in B) (n = 4 independent biological samples). **E-F)** Antigen transfer from OVA-647-loaded “donor” LPS-matured BMDCs labeled with CTV [E, D(CTV^+^OVA^+^)] or not [F, D(CTV^-^OVA^+^)] to “recipient” antigen-naïve mature BMDCs (n = 3 independent biological samples). **G-H)** FC analysis of G) surface CD86 costimulatory molecule and H) surface MHC-II maturation marker MFI levels. (CTRb/OVAb: n=3; CTR/OT-I-L/OT-I: n = 4 independent biological samples); **I)** Secreted IL-12p70 measured by ELISA (CTR/DMSO/Tg/pBr n=5/3/6/4 independent biological samples, duplicate wells). Bars represent means + SEM. Each dot represents one independent biological replicate, corresponding to the average of technical replicate wells where applicable. Significant p-values were calculated using two-way ANOVA with Tukey’s post-hoc tests in A and E-F comparing treatments to the corresponding controls within each antigen condition, one-way ANOVA with Tukey’s post-hoc tests in B-D, two-way ANOVA with Šídák’s post-hoc tests in G-H, and two-way ANOVA with Dunnett’s post-hoc tests in I. Exact p-values are indicated above bars when significant.

**Fig. S8. Flow cytometry gating strategy for the antigen transfer assay. A-E)** Gating strategy used to quantify antigen transfer between BMDCs. A-C) Common initial gating steps for all samples: **A)** total cells were selected based on forward and side scatter; **B)** singlets were gated based on FSC-H versus FSC-A; **C)** live cells were identified as DRAQ7-negative cells. **D-E)** Gating used to distinguish donor and recipient populations and quantify antigen transfer based on OVA-AF647 signal. **D)** In the condition where donor cells were CTV-negative and recipient cells were CTV-positive, donor cells (D) were identified as CTV⁻ and scored as OVA-AF647⁺ or OVA-AF647⁻, while recipient cells (R) were identified as CTV⁺ and scored as OVA-AF647⁺ (antigen transfer) or OVA-AF647⁻. **E)** In the reciprocal condition, where donor cells were CTV-positive and recipient cells were CTV-negative, donor cells (D) were identified as CTV⁺ and scored as OVA-AF647⁺ or OVA-AF647⁻, while recipient cells (R) were identified as CTV⁻ and scored as OVA-AF647⁺ (antigen transfer) or OVA-AF647⁻. **F-H)** Control samples used to define gating boundaries: **F)** CTV-positive cells only; **G)** CTV-negative cells only; **H)** OVA-AF647-positive, CTV-negative cells.

**Fig. S9 Viability, proliferation, and intracellular cytokine analysis of human MoDC:T cell co-cultures after SOCE modulator or ionomycin treatment. A)** LDH release from cytokine-matured MoDCs treated for 4 h with DMSO, Tg (0.1 µM), pBr (10 µM), or ionomycin (Iono, 0.1 µM), in the presence (blue) or absence (grey) of MLANA-L peptide, followed by overnight culture. Triton X-100 (TX) was used as a positive control for cell death. LDH release was calculated for each donor after background subtraction and normalized to the corresponding TX positive control, then expressed as percentage of total LDH release. n = 3 independent MoDC donors. **B-E)** Autologous co-cultures of cytokine-matured HLA-A2⁺ MoDCs and CTV-labeled total T cells were treated or not (CTR, grey) with DMSO (cyan), Tg (purple), pBr (yellow), or Iono (red) during peptide loading as in A, washed, and co-cultured for 48 h in the absence of antigen (NoAg), or in the presence of a long peptide pool (Mel-L) or an HLA-A2-compatible minimal peptide pool (Mel-S) derived from three melanoma-associated antigens, Melan-A, NY-ESO, and gp100. **B)** Proliferation of CD4⁺ and CD8⁺ T cells, measured as the percentage of cells showing CTV dilution relative to non-stimulated control cells. **C-E)** Intracellular cytokine staining of CD4⁺ T cells, CD8⁺ T cells, and MoDCs (CD11c⁺), showing the frequency of **C)** IL-2⁺, **D)** IFNG⁺, and **E)** IL-2⁺IFNG⁺ cells. Bars are means + SEM. Each dot represents one independent donor. Data in B-E are normalized to the mean donor response. For A, significant p-values were calculated using two-way ANOVA with Dunnett’s post-hoc test comparing each condition to the corresponding antigen-matched control; significant differences to TX are omitted for clarity. For B-E, significant p-values were calculated using two-way ANOVA with Tukey’s post-hoc test. Exact p-values are indicated above bars when significant. n = 4 independent donors for B-E.

**Fig. S10 Flow cytometry gating strategy for intracellular cytokine staining in human MoDC:T cell co-cultures. A-J)** Representative gating strategy for intracellular cytokine staining (ICS) and proliferation analysis in human MoDC:T cell co-cultures, shown using CD3/CD28 bead stimulation as a positive control. **A-B)** Cells were first selected based on forward and side scatter **A)**, and live cells were identified by exclusion of viability dye-positive cells in the CD3/viability plot **B)**.**C)** T cells and MoDCs were then distinguished based on CD3 and CD11c expression. **D)** T cells were further subdivided into CD4⁺ and CD8⁺ T cells. **E-F)** Within CD4⁺ T cells, intracellular IL-2 and IFNG expression **E)** and CTV dilution **F)** were analyzed. **G-H)** Within CD8⁺ T cells, intracellular IL-2 and IFNG expression **G)** and CTV dilution **H)** were analyzed. **I-J)** MoDCs were further gated on singlets **I)**, and intracellular IL-2 and IFNG expression was analyzed **J)**. **K-Q)** Control samples used to define gating boundaries.K) T-cell-only control used to define the MoDC gate and assess background/autofluorescence in the CD11c/CD3 gating space. **L)** Fluorescence-minus-one (FMO) control for IL-2 and IFNG, shown for CD8⁺ T cells; equivalent controls were used for CD4⁺ T cells. **M)** FMO control for IL-2 and IFNG, gated on MoDC singlets. **N-O)** CTV-labeled T cells cultured without MoDC co-culture, used to define non-proliferating or background CTV dilution gates in **N)** CD4⁺ and **O)** CD8⁺ T cells. **P-Q)** Unlabeled T cells cultured without MoDC co-culture, used as CTV-negative controls for **P)** CD4⁺ and **Q)** CD8⁺ T cells.

**Fig. S11. Pathway analysis and differential gene expression in MoDCs after SOCE modulator treatment.** Bulk RNA sequencing analysis of MoDCs treated with 0.1 µM Tg,10 µM pBr or untreated (CTR) for 6 h (see also Fig. 6 and Data S1). **A-B)** Over-representation and gene set enrichment analyses (ORA and fGSEA) were performed using multiple databases, including GO, KEGG, Reactome, WikiPathways, and Hallmark MSigDB, resulting in 1,055 significantly enriched pathways when comparing Tg treatment to CTR. **A)** Word cloud visualization of recurrent pathway terms. **B)** Schematic representation of the workflow used to classify enriched pathways into thematic categories. Briefly, word cloud visualization and keyword frequency analysis were used to identify recurrent terms, after which pathways were manually grouped based on these terms and biological relevance. These categories are summarized in Fig. 6B and in the “Pathway Summary” sheet of Data S1, with color-coded annotations applied across individual pathway analysis sheets to indicate category membership. **C)** Volcano plot (log_2_ fold-change, FC vs -log_10_ False Discovery Rate, FDR) highlighting significantly upregulated genes after pBr treatment (7 genes, dark red; log_2_FC > 1, FDR < 0.05). Light red, genes with log_2_FC > 1 that did not reach FDR < 0.05; blue, genes with log_2_FC < -1. **D)** Heatmap of significantly differentially expressed genes after pBr treatment. Each column within a treatment condition represents an independent donor. The color scale represents expression level (blue to red).

**Fig. S12. SOCE modulators alter cytokine secretion in MoDCs and BMDCs. A-B)** Cytokine array screening of supernatants collected after 4h treatment and overnight culture from cytokine-matured MoDC cultures, pooled from 3 different donors, left **A)** without antigen or **B)** loaded with MLANA-L peptide. Cultures were treated as indicated with Tg (0.1 µM), DMSO vehicle control, or no compound (CTR). Left: violin plots comparing the distribution of global array RLU values, including all cytokine spot values, across conditions. Right: dot blot array images for each treatment. See also Figure 6. **C-E)** Cytokine array screening of 4h supernatants from LPS-matured BMDC cultures, pooled from 3 different mice, loaded with OT-I-L or OVAb and treated with Tg (0.1 µM), pBr (5 µM), DMSO vehicle control, or left untreated (CTR). **C)** Heatmap representing normalized RLU values for OT-I-L- and OVAb-loaded BMDCs. Panels are divided into high, intermediate, and low expression levels for clarity. Arrows indicate cytokines upregulated by both Tg and pBr compared with either CTR or DMSO conditions. See also Supplementary Data S2. D-**E)** Violin plots and dot blot array images for **D)** OT-I-L- and **E)** OVAb-loaded BMDCs. Left: violin plots comparing the distribution of global array RLU values, including all cytokine spot values, across conditions. Right: dot blot array images for each treatment. Normality of the datasets was assessed using the Shapiro–Wilk test. Significant p-values were calculated using the Friedman test with Dunn’s post-hoc test and are indicated above the corresponding plots when significant..
